# Induction chemotherapy followed by camrelizumab plus apatinib and chemotherapy as first-line treatment for extensive-stage small-cell lung cancer: a multicenter, single-arm trial

**DOI:** 10.1038/s41392-025-02153-7

**Published:** 2025-02-18

**Authors:** Ming Liu, Guihuan Qiu, Wenhui Guan, Xiaohong Xie, Xinqing Lin, Zhanhong Xie, Jiexia Zhang, Yinyin Qin, Haijian Du, Xin Chen, Yu Deng, Shiyue Li, Nanshan Zhong, Chengzhi Zhou

**Affiliations:** 1 State Key Laboratory of Respiratory Disease, National Clinical Research Center for Respiratory Disease, National Center for Respiratory Medicine, Department of Pulmonary and Critical Care Medicine, Guangzhou Institute of Respiratory Health, The First Affiliated Hospital of Guangzhou Medical University, Guangzhou, China; 2 Minimally Invasive Cancer Treatment Center, Guangdong Second Provincial General Hospital, Guangzhou, China; 3 Department of Pulmonary and Critical Care Medicine, Zhujiang Hospital, Southern Medical University, Guangzhou, China; 4 Department of Radiology, The First Affiliated Hospital of Guangzhou Medical University, Guangzhou, China

**Keywords:** Cancer, Biomarkers

## Abstract

Chemo-immunotherapy is the current first-line treatment for patients with extensive-stage small cell lung cancer (ES-SCLC), but survival benefits are modest. We aimed to evaluate the safety, antitumor activity and biomarkers of first-line camrelizumab and apatinib plus chemotherapy in untreated ES-SCLC patients. In this single-arm trial (ClinicalTrials.gov NCT05001412), eligible patients received 2 cycles of etoposide and carboplatin (EC) as induction treatment followed by 2–4 cycles of camrelizumab, apatinib plus EC, then maintenance camrelizumab plus apatinib. Primary endpoint was safety. Secondary endpoints included objective response rate (ORR), duration of response, progression-free survival (PFS), and overall survival (OS). Targeted sequencing and whole transcriptome sequencing were performed to explore biomarkers. All enrolled 40 patients were treated and analyzed for safety. During the entire treatment, treatment-emergent adverse events (TEAEs) occurred in 40 patients (100%), and 30 (75.0%) were grade ≥3. The most common grade ≥3 TEAEs were neutropenia (35.0%), anemia (15.0%) and increased alanine aminotransferase (15.0%). No treatment-related deaths occurred. Among 36 evaluable patients, ORR was 88.9% (95% CI: 73.9%–96.9%), median PFS was 7.3 months (95% CI: 6.6–9.2) and median OS was 17.3 months (11.8-not reached). Mutations in RB1, high levels of tumor mutation burden, natural killer cells, and interferons, and low levels of cancer-associated fibroblasts, correlated with prolonged PFS. Induction chemotherapy followed by camrelizumab, apatinib plus EC demonstrated acceptable safety and promising antitumor activity in untreated ES-SCLC patients. The identified biomarkers need further validation.

**Trial Registration** ClinicalTrials.gov Identifier: NCT05001412.

## Introduction

Small-cell lung cancer (SCLC) is an aggressive and rapidly progressing subtype of lung cancer, comprising about 15% of all lung cancer diagnoses.^1^ SCLC is associated with a dismal prognosis, with a median overall survival (OS) limited to just 7 months.^2^ At diagnosis, approximately 70% of SCLC patients are found to have extensive-stage disease (ES-SCLC).^3^ In 2019, the FDA approved a chemo-immunotherapy regimen combining platinum-based chemotherapy and the PD-L1 inhibitor atezolizumab, based on the results from the IMpower133 trial.^4^ This marked a significant step forward in the treatment landscape of ES-SCLC and established chemo-immunotherapy as the standard of care for newly diagnosed ES-SCLC patients. Subsequent phase 3 trials also substantiated the clinical benefits of incorporating PD-L1 inhibitors into first-line regimens, demonstrating improved outcomes in this aggressive disease.^5–8^ Despite these advancements, the survival improvements are modest, with median OS extending by just 2.0 to 4.7 months.^5–8^ Hence, novel therapies are urgently needed to further improve outcomes for patients with ES-SCLC.

Recent studies have revealed the highly heterogeneous and immunosuppressive microenvironment of SCLC, and insufficient CD8 + T cell infiltration is a key reason for limited response to immune checkpoint inhibitors (ICIs) in SCLC.^9^ The aggressive growth and invasiveness of SCLC are driven by angiogenesis, and vascular endothelial growth factor (VEGF) overexpression correlates with poor prognosis in patients with SCLC.^10,11^ The overexpressed VEGF downregulates endothelial adhesion molecules, like ICAM-1 and VCAM-1, thereby reducing immune cell adhesion and migration.^12^ Thus, targeting the VEGF pathway might increase CD8 + T cells infiltration and reduce neovascularization, potentially enhancing the antitumor response in ES-SCLC. Combining ICIs with anti-VEGFR agents has synergistic effects via increasing T-cell infiltration in the tumor microenvironment.^13^ Preclinical data have shown that apatinib, a VEGFR inhibitor, can modulate the tumor microenvironment by decreasing tumor hypoxia, enhancing CD8 + T cell infiltration, and decreasing the accumulation of tumor-associated macrophages in lung cancer tissues.^14^ In lung cancer mouse models, the combination of apatinib with anti-PD-L1 antibodies led to significant suppression of tumor growth and metastasis while prolonging mouse survival.^14^

The combination strategy of ICIs, anti-VEGFR agents, and chemotherapy has shown promising efficacy in patients with lung cancer. Specifically, in the IMpower 150 trial, first-line therapy with atezolizumab plus bevacizumab and platinum-based chemotherapy notably improved OS (median OS: 19.2 months versus 14.7 months; hazard ratio [HR] = 0.78) and objective response rate (ORR; 63.5% versus 48.0%) compared with bevacizumab combined with platinum-based chemotherapy in patients with non-small cell lung cancer (NSCLC).^15^ Additionally, the incorporation of an anti-VEGFR agent, bevacizumab, into cisplatin and etoposide also enhanced progression-free survival (PFS; median PFS: 6.7 months versus 5.7 months; HR = 0.72) and ORR (55.3% versus 58.4%; odds ratio=1.13) compared with cisplatin and etoposide alone in newly diagnosed ES-SCLC patients.^16^ Recently, the ETER 701 trial demonstrated the efficacy of combining ICIs, anti-VEGFR agents, and platinum-based chemotherapy in ES-SCLC patients in the first-line setting.^17^ This combination regimen, comprising benmelstobart, anlotinib, carboplatin, and etoposide significantly prolonged OS (median OS: 19.3 months versus 11.9 months; HR = 0.61) and increased ORR (81.3% versus 66.8%) compared with carboplatin and etoposide alone in patients with ES-SCLC.^17^ However, in the clinical setting, most SCLC cases are centrally located near the hilum and large blood vessels,^2^ which carry a high risk of bleeding. Previous trials of anti-VEGFR agent combinations for SCLC typically excluded patients with large vessel invasion or high bleeding risk.^17,18^ This exclusion poses a challenge to the clinical application or optimization of anti-angiogenic combination strategies in this subset of ES-SCLC patients. Addressing this gap is essential, as these patients represent a considerable proportion of the ES-SCLC population.

Apatinib, a VEGFR2-targeting tyrosine kinase inhibitor, and camrelizumab, a PD-1 inhibitor, has each shown notable antitumor activity in ES-SCLC patients beyond the first-line treatment.^19,20^ The PASSION study reported that camrelizumab plus apatinib yielded promising antitumor activity and was well-tolerable in ES-SCLC patients, including those with responsive and resistant to chemotherapy, in the second-line setting.^21^ A retrospective study showed that first-line camrelizumab plus chemotherapy, then maintenance camrelizumab plus apatinib, provided better survival benefits compared with PD-L1 inhibitors plus chemotherapy and exhibited strong antitumor activity.^22^ At the ASCO 2024 meeting, this combination also showed efficacy (ORR: 82.14%; median PFS: 7.56 months) and tolerable safety in untreated ES-SCLC patients.^18^ In this study, we aimed to evaluate the safety and antitumor activity of first-line induction etoposide and carboplatin (EC), followed by a combination of camrelizumab, apatinib, and EC in ES-SCLC patients, including those with large vessel invasion. The choice of induction chemotherapy was based on its high ORR of 97% after 2 treatment cycles in limited-disease SCLC.^23^ This suggests that for most patients, this approach could promote tumor shrinkage and separation from surrounding vasculature, thereby reducing the bleeding risk associated with anti-VEGFR agents. Moreover, this approach could induce immunogenic cell death, thereby enhancing the efficacy of subsequent immunotherapy.^24^ When we designed this study, chemotherapy alone was also the standard recommended first-line regimen for patients with ES-SCLC.^25^ Concurrently, we identify potential biomarkers that could predict clinical response.

## Results

### Patients and treatment

**Fig. 1:**
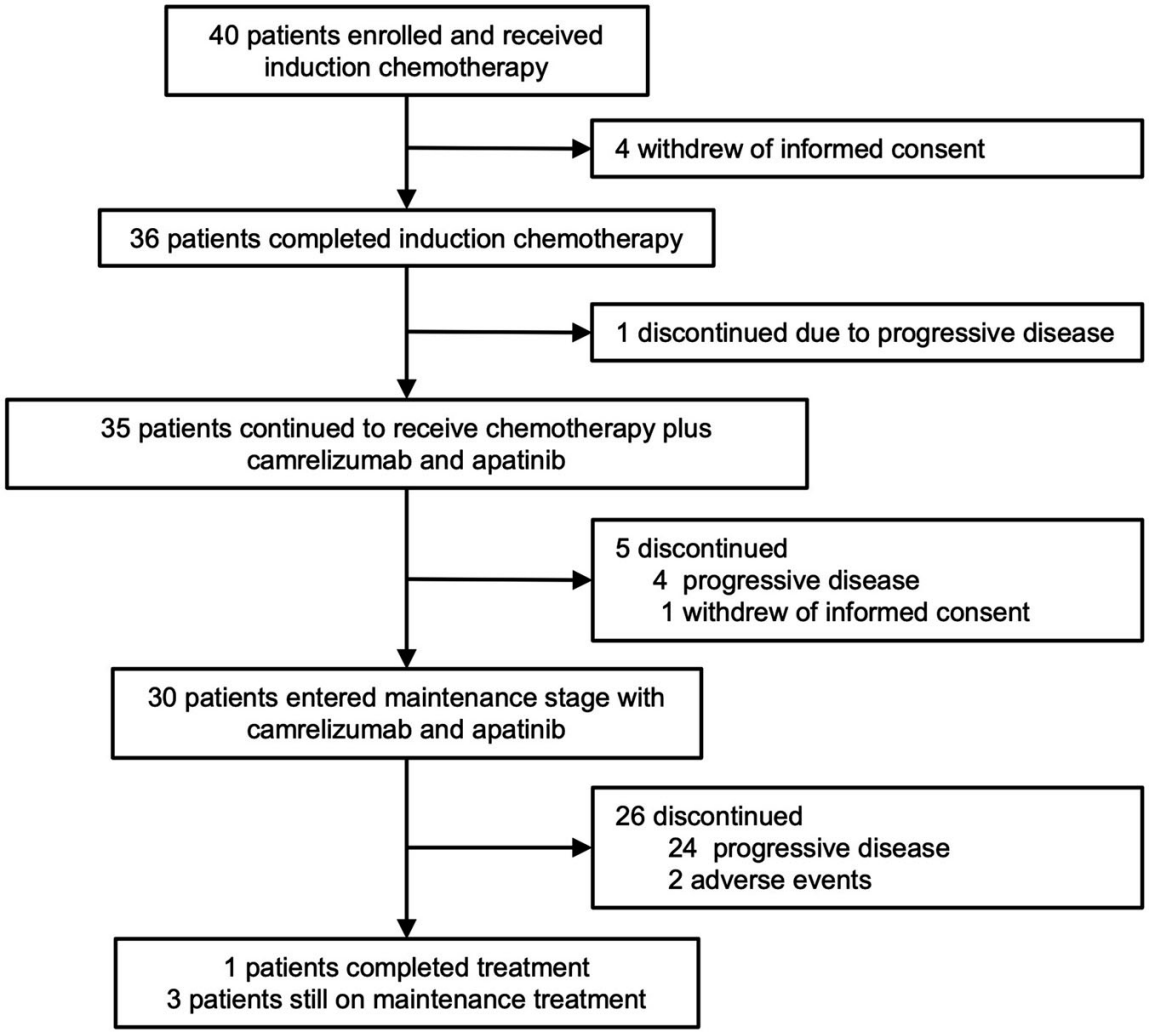
Patients flow chart

Between 21 January 2021 and 20 August 2022, 40 patients were included and received induction EC (Fig. 1). After one cycle of induction EC, 4 patients withdrew informed consent and were unevaluable for tumor response. Thus, 40 patients were evaluable for safety, and 36 patients were evaluable for tumor response. Of 40 patients, the median age was 60 years (range: 40-73), and 36 (90.0%) were male. All 40 patients (100%) presented with stage IV disease. Most patients had central SCLC (33/40, 82.5%) and had an Eastern Cooperative Oncology Group (ECOG) performance status (PS) of 1 (31/40, 77.5%). Table 1 presents the baseline characteristics. Vascular invasion was assessable in 35 patients, and all of them had large vessel invasion (Table 1, Supplementary Table 1).

**Table 1:** Baseline characteristic

Characteristics	All patients (*n* = 40)
Age, years, median (range)	60 (40–73)
< 65	29 (72.5)
≥ 65	11 (27.5)
Male	36 (90.0)
Eastern Cooperative Oncology Group PS	
0	9 (22.5)
1	31 (77.5)
Smoking status	
Never	2 (5.0)
Current/Former	38 (95.0)
Basic disease	
Chronic obstructive pulmonary disease	16 (40.0)
Hepatitis B	2 (5.0)
Hemoptysis	1 (2.5)
Others	21 (52.5)
Anatomical type	
Central	33 (82.5)
Peripheral	7 (17.5)
Vascular invasion grade	
Grade 1	6 (15.0)
Grade 3	29 (72.5)
NA	5 (12.5)
Disease stage	
IV	40 (100.0)
Brain metastases	
Yes	6 (15.0)
No	34 (85.0)
Liver metastases	
Yes	7 (17.5)
No	33 (82.5)
Number of metastatic organs	
1	17 (42.5)
≥ 2	23 (57.5)
dNLR	
≤ 3	32 (80.0)
> 3	8 (20.0)
Lactate dehydrogenase	
≤ Upper limit of normal	16 (40.0)
> Upper limit of normal	19 (47.5)
Unknown	5 (12.5)

Data are n (%). *dNLR* derived neutrophil-to-lymphocyte ratio, *NA* not assessable

In total, 36 patients completed initial 2 cycles of induction EC. Subsequently, one patient discontinued treatment due to disease progression. Thus, 35 patients received camrelizumab plus apatinib plus EC. During this phase, additional 5 patients discontinued treatment due to progressive disease (*n* = 4) and withdrawal of informed consent (*n* = 1). Finally, 30 patients entered the maintenance treatment phase with camrelizumab plus apatinib. The reasons for treatment discontinuation during maintenance treatment were progressive disease (*n* = 24) and adverse events (*n* = 2).

At the data cut-off on May 30, 2023, the median duration of follow-up was 20.6 months (range: 4.1–27.5). 3 (8.3%) of 36 patients were still on the study treatment. 30 patients (83.3%) received 6 cycles of EC (Supplementary Table 2). The median treatment cycles of camrelizumab were 7.0 (range: 2.0–35.0). The median treatment duration of apatinib was 5.23 months (range: 0–24.2). The reasons for not completing 4 cycles of camrelizumab plus apatinib plus EC are provided in Supplementary Table 3.

### Safety

During the entire treatment phase, any grade treatment-emergent adverse events (TEAEs) occurred in 40 patients (100%), with the most common being leukopenia (31 [77.5%]), anemia (28 [70.0%]), and neutropenia (25 [62.5%]) (Table 2). Grade 3/4 TEAEs occurred in 30 patients (75.0%), with the most common being neutropenia (14 [35.0%]), anemia (6 [15.0%]), and increased alanine aminotransferase (6 [15.0%]). No deaths were considered related to study drug by the investigator. Serious adverse events occurred in 6 (15.0%) patients.

**Table 2:** Treatment-emergent adverse events occurred in at least 10% of patients during the entire treatment phase (*n* = 40)

	Any grade	Grade 1	Grade 2	Grade ≥ 3
Any treatment-emergent adverse events	40 (100.0)	37 (92.5)	38 (95.0)	30 (75.0)
Leukopenia	31 (77.5)	6 (15.0)	22 (55.0)	3 (7.5)
Anemia (Lower hemoglobin)	28 (70.0)	12 (30.0)	10 (25.0)	6 (15.0)
Neutropenia	25 (62.5)	4 (10.0)	7 (17.5)	14 (35.0)
Thrombocytopenia	17 (42.5)	6 (15.0)	6 (15.0)	5 (12.5)
Cough	14 (35.0)	7 (17.5)	7 (17.5)	0
Hyperthyroidism	13 (32.5)	13 (32.5)	0	0
Fatigue	13 (32.5)	8 (20.0)	5 (12.5)	0
Hand foot syndrome	13 (32.5)	5 (12.5)	7 (17.5)	1 (2.5)
Hair loss	13 (32.5)	11 (27.5)	2 (5.0)	0
Increased alanine aminotransferase	10 (25.0)	3 (7.5)	1 (2.5)	6 (15.0)
Increased aspartate aminotransferase	9 (22.5)	2 (5.0)	3 (7.5)	4 (10.0)
Dizziness	9 (22.5)	6 (15.0)	3 (7.5)	0
Increased thyroid stimulating hormone	7 (17.5)	5 (12.5)	2 (5.0)	0
Hypokalemia	7 (17.5)	6 (15.0)	1 (2.5)	0
Hyponatremia	7 (17.5)	1 (2.5)	1 (2.5)	5 (12.5)
Diarrhea	7 (17.5)	4 (10.0)	3 (7.5)	0
Bloating	7 (17.5)	5 (12.5)	2 (5.0)	0
Hemoptysis	7 (17.5)	4 (10.0)	3 (7.5)	0
Hypertension	6 (15.0)	0	1 (2.5)	5 (12.5)
Dyspnea	6 (15.0)	3 (7.5)	3 (7.5)	0
Influenza (COVID-19)	6 (15.0)	6 (15.0)	0	0
Headache	6 (15.0)	6 (15.0)	0	0
Flu-like symptoms	5 (12.5)	5 (12.5)	0	0
Immune-mediated hepatitis	5 (12.5)	2 (5.0)	2 (5.0)	1 (2.5)
Elevated blood lactate dehydrogenase	5 (12.5)	5 (12.5)	0	0
Anorexia	5 (12.5)	4 (10.0)	1 (2.5)	0
Cancer pain	4 (10.0)	2 (5.0)	2 (5.0)	0
Constipate	4 (10.0)	3 (7.5)	1 (2.5)	0
Nausea	4 (10.0)	1 (2.5)	3 (7.5)	0

Data are *n* (%)

Any grade immune-related adverse events (irAEs) occurred in 30 patients (85.7%) and grade 3/4 irAEs occurred in 9 patients (25.7%). The most common irAEs were hyperthyroidism (13 [37.1%]), increased alanine aminotransferase (8 [22.9%]), and increased thyroid stimulating hormone (7 [20.0%]) (Supplementary Table 4). Adverse events (AEs) related to apatinib are presented in Supplementary Table 5. No grade ≥ 3 bleeding events related to apatinib were observed.

AEs led to treatment discontinuation of camrelizumab in 4 patients (11.4%) and apatinib in 2 patient (5.7%), including grade 3 pneumonitis, grade 3 hyponatremia, grade 2 muscle spasm, and grade 2 pulmonary tuberculosis (*n* = 1 each). No AEs led to treatment discontinuation of chemotherapy. AEs led to treatment delay of camrelizumab in 12 patients (34.3%) and treatment interruption of apatinib in 7 patients (20.0%). AEs led to dose reduction of apatinib in 2 patients (5.7%) and chemotherapy in 3 patients (7.5%).

### Efficacy

After 2 cycles of induction EC, 24 of 36 patients had an objective response (66.7%, 95% CI: 49.0–81.4); 35 of 36 patients had disease control (97.2%, 95% CI: 85.5–100) (Table 3). Among them, 24 (66.7%) patients had partial response (PR), and 11 (30.6%) had stable disease (SD). Notably, 72.7% (8/11) of those with initial SD then achieved PR after combination treatment with camrelizumab, apatinib plus EC, followed by maintenance camrelizumab plus apatinib.

**Table 3:** Antitumor response

	Patients (*n* = 36)
Response to induction treatment	
ORR	24 (66.7, 49.0–81.4)
DCR	35 (97.2, 85.5–100)
Overall response	
ORR	32 (88.9, 73.9–96.9)
Confirmed ORR	28 (77.8, 60.8–89.9)
DCR	35 (97.2, 85.5–100)
Best overall response	
Partial response	32 (88.9, 73.9–96.9)
Stable disease	3 (8.3, 1.8–22.5)
Progressive disease	1 (2.8, 0.7–14.5)

Data are n (%, 95% CI). *ORR* objective response rate, *DCR* disease control rate. Antitumor response was assessed in patients who had at least one post-treatment tumor evaluation per RECIST v1.1

**Fig. 2:**
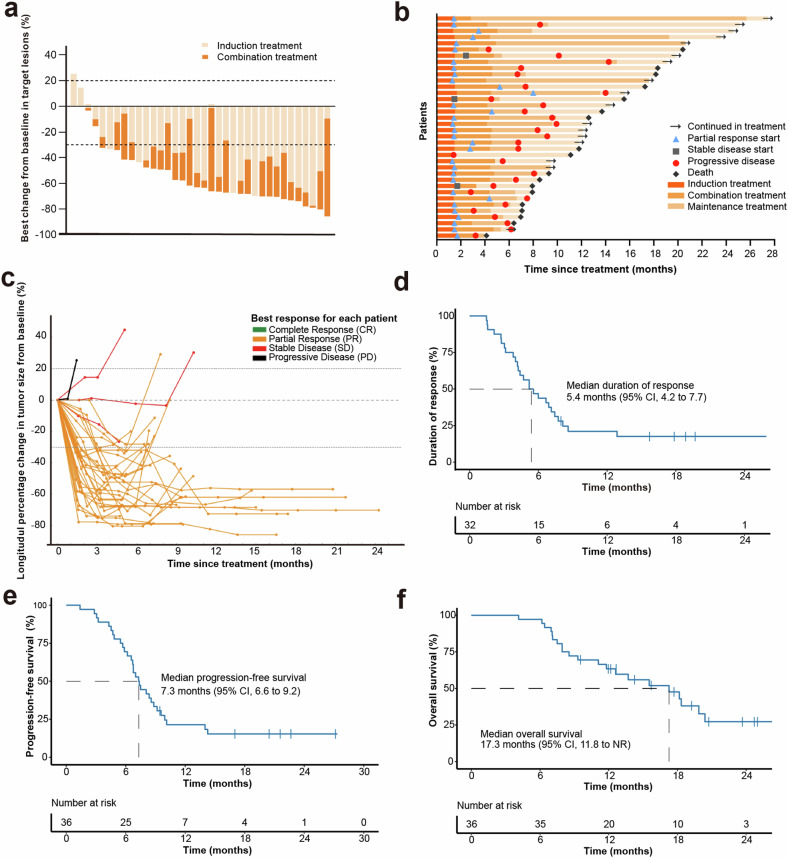
Clinical outcomes. **a** Maximum reduction from baseline in target lesion. **b** Treatment response and duration. **c** Spider plot showing the percentage change in the sum of target lesion diameters during treatment. **d** Duration of response. **e** Kaplan-Meier curve for progression-free survival. **f** Kaplan-Meier curve for overall survival. NR not reached

After the entire treatment, 32 of 36 patients had an objective response (88.9%, 95% CI: 73.9–96.9); 35 of 36 patients had disease control (97.2%, 95% CI: 85.5–100) (Table 3). Among them, 32 (88.9%) had PR, and 3 (8.3%) had SD. In total, 34 patients (94.4%) had a decrease in tumor size of target lesions from the baseline. Median best change from baseline was −63.3% (Fig. 2a). Median duration of response (DoR) was 5.4 months (95% CI: 4.2–7.7; Fig. 2b–d). Median time to response (TTR) was 1.5 months. The overall ORR in all 40 patients was provided in the Supplementary Table 6.

At the data cut-off on May 30, 2023, the median duration of follow-up was 20.6 months (range: 4.1–27.5). Of the 36 evaluable patients, 30 (83.3%) had disease progression or deaths (*n* = 21). The median PFS was 7.3 months (95% CI: 6.6–9.2, Fig. 2e). The median OS was 17.3 months (95% CI: 11.8-NR). The 12-month OS rate was 63.4% (95% CI: 45.4%–76.9%) (Fig. 2f).

### Exploratory biomarkers

**Fig. 3:**
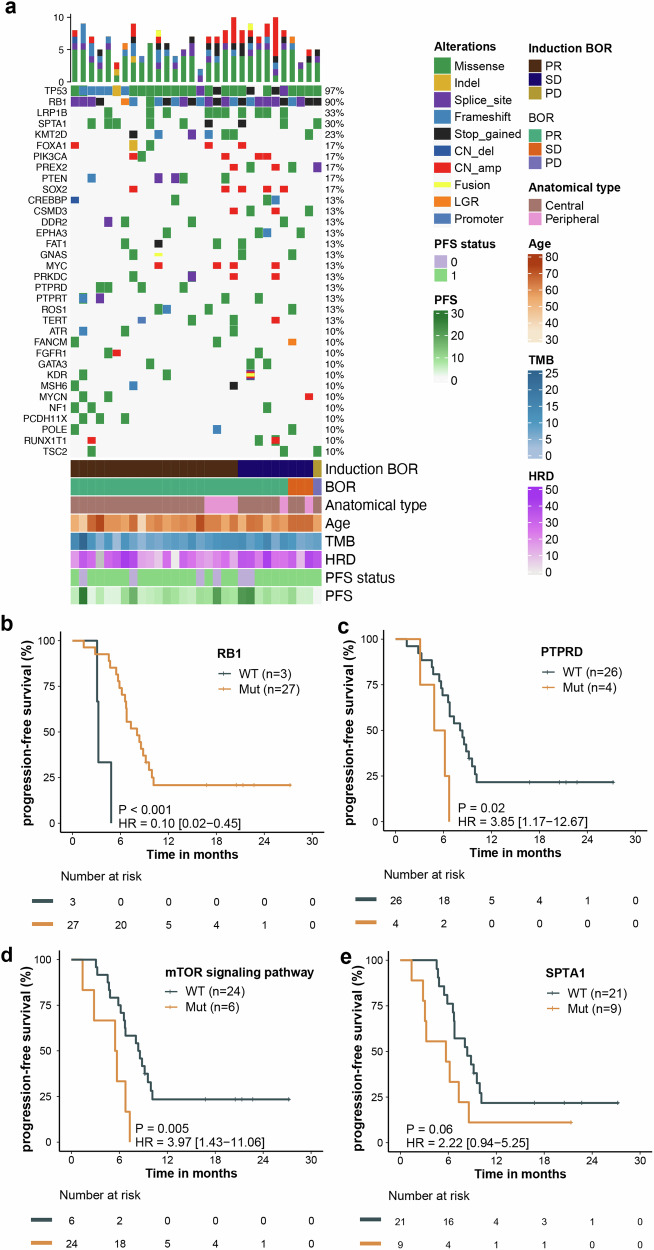
Response to treatment based on genomic alterations. **a** Distribution of genetic alterations and association with treatment response. **b**–**e** Association between PFS and mutations in RB1 (**b**), PTPRD (**c**), mTOR signaling pathway (**d**) and SPTA1 (**e**). BoR best of response, TMB tumor mutation burden, HRD homologous recombination deficiency, PFS progression-free survival

We evaluated the association between genomic alteration and clinical outcomes. Baseline tissue samples were available from 30 patients for targeted gene sequencing and from 21 patients for whole transcriptome sequencing (WTS). TP53 (97%) and RB1 (90%) were the most frequently mutated genes (Fig. 3a). No significant correlation between genomic mutations and response (complete response/PR) during the induction treatment and the entire treatment was observed. Mutations in RB1 were associated with longer PFS (*P* < 0.001; HR, 0.10; 95% CI: 0.02–0.45), whereas mutations in PTPRD (*P* = 0.02; HR, 3.85; 95% CI: 1.17–12.67) and the mTOR signaling pathway genes (*P* = 0.005; HR, 3.97; 95% CI: 1.43–11.06) were associated with shorter PFS (Fig. 3b–d). A trend toward shorter PFS was observed in patients with mutations in SPTA1 (P = 0.06; HR, 2.22; 95% CI: 0.94–5.25) (Fig. 3e).

**Fig. 4:**
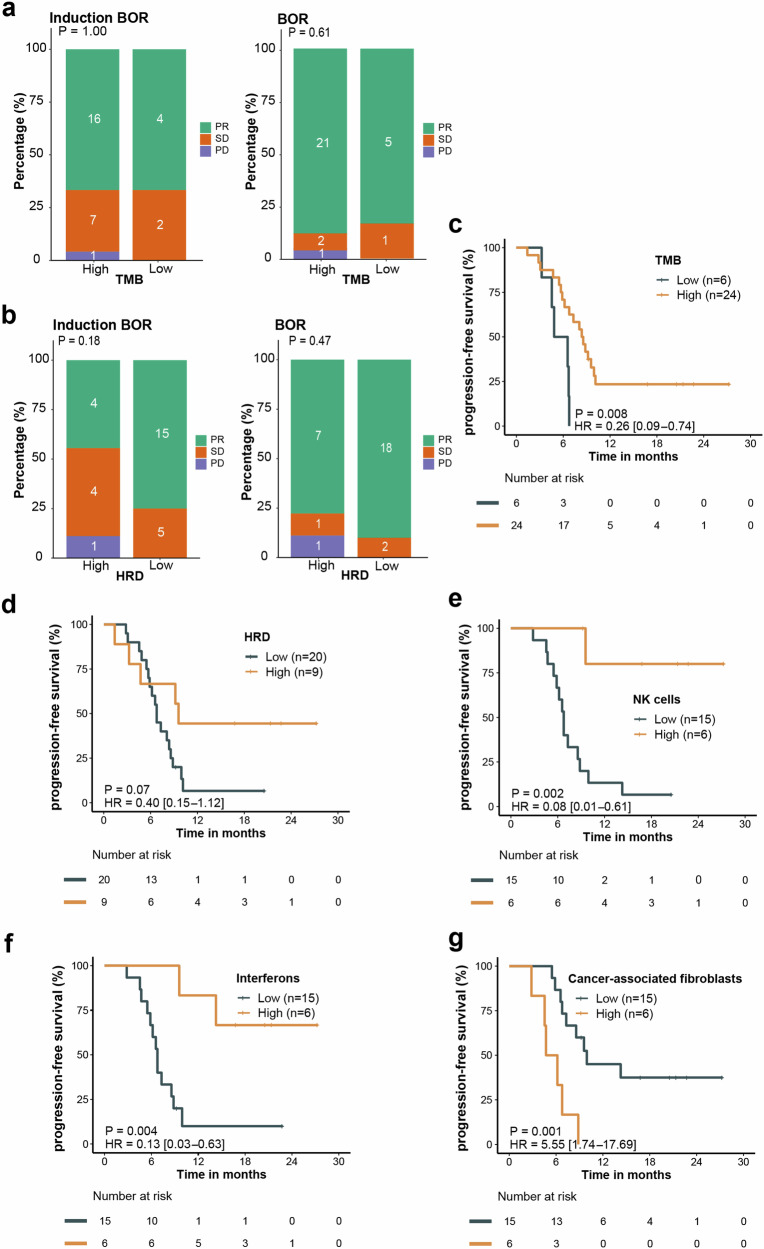
Response to treatment based on TMB, HRD and tumor microenvironment status. **a**, **b** BoR based on TMB status (**a**) and HRD score (**b**). **c**, **d** Kaplan-Meier curves of PFS based on TMB (**c**) and HRD score (**d**). **e**–**g** Kaplan-Meier curves of PFS based on NK cell value (**e**), interferons expression (**f**) and cancer-associated fibroblasts value (**g**). PFS, progression-free survival, BoR best of response, TMB tumor mutation burden, HRD homologous recombination deficiency, NK natural killer

We observed that tumor mutation burden (TMB) status and homologous recombination deficiency (HRD) score did not significantly correlate with response (complete response/PR) during the induction treatment and the entire treatment (Fig. 4a, b). High TMB (TMB ≥ 7.0) correlated with longer PFS (*P* = 0.008; HR, 0.26; 95% CI: 0.09–0.74) (Fig. 4c). Patients with high HRD score (HRD ≥ 34.0) had trend toward longer PFS (*P* = 0.07; HR, 0.40; 95% CI: 0.15–1.12) (Fig. 4d). Immune cell infiltration analysis revealed that high levels of NK cells (*P* = 0.002; HR, 0.08; 95% CI: 0.01–0.61) and interferons (*P* = 0.004; HR, 0.13; 95% CI: 0.03–0.63) correlated with longer PFS, whereas high cancer-associated fibroblasts levels correlated with shorter PFS (*P* = 0.001; HR, 5.55; 95% CI: 1.74–17.69) (Fig. 4e–g).

## Discussion

As far as we are aware, this is the first report to investigate the safety, antitumor response and potential biomarkers of PD-1 inhibitors plus anti-VEGFR agents and chemotherapy in ES-SCLC patients. In this single-arm trial, induction EC followed by a combination of camrelizumab, apatinib and EC, and subsequent maintenance with camrelizumab plus apatinib, had a manageable safety profile in untreated ES-SCLC patients. Notably, this regimen showed promising antitumor activity, with ORR of 88.9% and disease control rate (DCR) of 97.2%. Median PFS was 7.3 months and median OS was 17.3 months. Our findings offered valuable insights for future clinical trials incorporating chemo-immunotherapy with anti-VEGFR agents in ES-SCLC.

The safety profile observed for our combination regimen of EC, camrelizumab, and apatinib aligns with previously reported toxicity profiles for these agents as monotherapies. We did not observe unexpected toxicities. The most common grade ≥3 TEAEs were leukopenia, anemia and increased alanine aminotransferase, which were mostly chemotherapy related. Most toxicities were manageable with dose adjustments and supportive care. Given that most cases of SCLC were centrally located and near major blood vessels, the use of anti-angiogenic agents could increase the risk of bleeding.^2,26^ In this study, 82.5% of patients enrolled were central ES-SCLC, and all patients evaluable for vascular invasion had large vessel invasion. However, no grade ≥ 3 bleeding events were observed. These findings suggest that first-line combination of EC, camrelizumab, and apatinib was tolerable in ES-SCLC patients.

The IMpower133 and CASPIAN studies reported that first-line atezolizumab or durvalumab plus EP had ORRs of 60.2% and 79%, median PFS of 5.2 and 5.1 months, and median OS of 12.3 and 13.0 months, respectively.^5,6^ The CAPSTONE-1 and ASTRUM-005 studies reported that adebrelimab or serplulimab plus EC had ORRs of 70.4% and 80.2%, median PFS of 5.8 and 5.8 months, and median OS of 15.3 and 15.4 months, respectively.^7,8^ A retrospective analysis showed that first-line camrelizumab plus EP/EC had an ORR of 65.7%, DCR of 74.3%, median PFS of 7.4 months, and median OS of 12.5 months.^27^ In this study, the ORR was 88.9%, with a DCR of 97.2%, median PFS of 7.3 months, and median OS of 17.3 months. Moreover, this study consisted of patients with more severe disease, all at stage IV. These results suggest that the combination provides favorable data over first-line chemotherapy plus ICIs in ES-SCLC, despite potential confounding factors from crossover trial comparisons such as study design, patient selection and chemotherapy regimens. This is consistent with high antitumor response reported for such a combination in SCLC and other solid tumors.^17,28–31^ This also aligns with evidence that anti-VEGFR agents can synergically improve the efficacy of immunotherapy in cancer treatment.^32^ Overall, combining chemo-immunotherapy with anti-VEGFR agents may enhance clinical outcomes for patients with previously untreated ES-SCLC.

Due to bleeding concerns, studies like the ETER 701 trial on anti-angiogenic drug combinations typically excluded patients with large vessel invasion.^17^ This exclusion challenges the optimization of therapeutic strategies for ES-SCLC with large vessel invasion, highlighting the need for broader inclusion criteria in clinical trials. Unlike the ETER701 trial, we used induction chemotherapy before the combination therapy to reduce bleeding events. At baseline, all 35 evaluable patients had large vessel invasion and were at high risk of bleeding. Nonetheless, the incidence of hemoptysis was similar to that reported in the ETER701 trial,^17^ with no grade ≥3 hemoptysis or other severe bleeding events. Moreover, our regimen achieved clinical outcomes comparable to benmelstobart and anlotinib plus EC in the ETER701 trial (ORR: 81.3%; median PFS: 6.9 months).^17^ These findings suggest that induction chemotherapy combined with camrelizumab and apatinib demonstrates promising efficacy in ES-SCLC. Only one patient experienced PD during the 2 cycles of induction chemotherapy, suggesting a low risk of PD during extended induction therapy. However, given the limited patient number, the risk-benefit of induction therapy in ES-SCLC requires further investigation. Overall, this study provides important data for future research to broaden the population eligible for first-line anti-angiogenic combinations in ES-SCLC.

Consistent with the ETER701 trial and other anti-angiogenesis, immunotherapy, and chemotherapy combination trial,^17,31^ disease progression was the main reason for treatment discontinuation in this study. This suggests that despite the clinical benefits of such a combination, most ES-SCLC patients may develop therapeutic resistance. Thus, new first-line treatments for ES-SCLC are still needed to overcome therapeutic resistance. Additionally, given that 82.8% (24/29) of disease progression occurred during the maintenance phase, more robust maintenance treatments may be feasible.

Currently, predictive biomarkers for ICIs in SCLC remain lack. The CASPIAN study demonstrated that PD-L1 expression level, a predictive biomarker for ICIs in various cancers, did not correlate with response to ICIs in SCLC.^5^ Only about 25% of SCLC patients had PD-L1 expression on ≥ 1% on tumor cells.^33^ This necessitates the exploration for new biomarkers. Our study identified several candidate predictive biomarkers via targeted sequencing and WTS. The most frequent mutation occurred in TP53 and RB1, consisting with prior reports.^34^ Previous studies indicated that TMB seemed to correlate with clinical activity in patients treated with single agent immunotherapy (nivolumab monotherapy) or PD-1 plus CTLA-4 blockage (nivolumab plus ipilimuma), but not for chemo-immunotherapy combinations in ES-SCLC.^6,35,36^ In the current study, patients with high TMB had better survival outcomes. These controversial results may be due to small sample size, the lack of standardized assays and score methods. The role of TMB in predicting chemo-immunotherapy in SCLC remains to be determined and warrants further investigation. Additionally, our analysis revealed longer PFS in patients with high NK cells, interferons, and RB1 mutations, whereas high cancer-associated fibroblasts levels and mutations in the mTOR signaling pathway and PTPRD were indicators of shorter PFS. The positive correlation between NK cells levels and PFS aligns with previous findings in ES-SCLC patients treated with first-line chemo-immunotherapy.^37^ However, the negative correlation between mutations in the mTOR signaling pathway and PTPRD and PFS differs from previous studies, which reported that mutations in the mTOR signaling pathway and PTPRD correlated with longer PFS in NSCLC patients treated with chemo-immunotherapy.^38,39^ These discrepancies may be due to the small sample size or different treatment regimens. Given the significant heterogeneity and the complex regulation of tumor immune microenvironment in SCLC, single biomarker seems inadequate to accurately predict the efficacy of chemo-immunotherapy. This underlines the need to develop predictive models and monitor biomarker dynamics during treatment in the further studies. Altogether, the identified biomarkers may help guide patient selection for camrelizumab plus apatinib and chemotherapy. However, further validation studies are needed before clinical application.

This study has several limitations. First, our study provides novel and valuable data into the safety and clinical activity of platinum-based chemotherapy combined with camrelizumab and apatinib plus as first-line regimen for ES-SCLC. However, the study design was not randomized with no comparative groups; thus, the antitumor activity was preliminary and cannot be analyzed for causality. Further randomized clinical studies are warranted to confirm the exact contribution of adding anti-VEGFR agents. Second, although incorporating biomarker analysis, limited availability of tissue sample warrants cautious interpretation of these results. Future studies of camrelizumab and apatinib plus chemotherapy in ES-SCLC, including body fluids biomarkers, are expected to build on our findings.

In conclusion, induction EC followed by a combination of camrelizumab, apatinib and EC, and subsequent maintenance camrelizumab plus apatinib, showed a tolearble safety and promising antitumor activity, suggesting its potential as a first-line therapy option for patients with ES-SCLC. Identified predictive biomarkers including gene mutations, TMB, and tumor microenvironment components.

## Methods and materials

The present study was approved by the ethical committee of the First Affiliated Hospital of Guangzhou Medical University (Guangzhou, China, No. 2020-189, 2021-1-13). The study was performed in accordance with the Declaration of Helsinki and Good Clinical Practice Guidelines. Written informed consent was provided by every participant before study entry. This trial was registered on ClinicalTrials.gov (NCT05001412).

### Patients

In this multicenter, single-arm study, enrolled patients were aged 18-75 years with histologically confirmed ES-SCLC who were treatment-naïve for ES-SCLC. Prior radiotherapy and chemotherapy for limited-stage SCLC were acceptable if a minimum of 6 months interval had elapsed between the last treatment and the diagnosis of ES-SCLC. Key inclusion criteria were an ECOG PS of 0 or 1, measurable lesions per RECIST v1.1, a minimum life expectancy of 12 weeks, and normal organ functionality. Patients were eligible if they were previously treated with apatinib, anti-PD-1 or anti-PD-L1 therapies. Patients with asymptomatic brain metastases not requiring immediate radiotherapy were permitted to participate.

### Procedures

Eligible patients received 2 cycles of induction etoposide (100 mg/m^2^, IV, on days 1 to 3) and carboplatin (AUC 5 mg/mL/min, IV, on day 1), then 2-4 cycles of camrelizumab (200 mg, IV, on day 1) and apatinib (250 mg, orally, daily) plus etoposide (100 mg/m^2^, IV, on days 1 to 3) and carboplatin (AUC 5 mg/mL/min, IV, on day 1). Patients continued maintenance treatment with camrelizumab (200 mg, IV, on day 1) and apatinib (250 mg, orally, daily) until disease progression, intolerable toxicity, death, consent withdrawal or up to 24 months. Each treatment cycle was 3 weeks.

Safety was evaluated at each treatment cycle. AEs were graded according to CTCAE v5.0 and reported from the day of informed consent signing until 30 days post the final study drug dose. Tumor response was assessed by investigators according to RECIST v1.1, using computed tomography or magnetic resonance imaging. Assessments were conducted at baseline, on day 21 of cycles 1 and 2, and then every 2 cycles until disease progression or the start of a new treatment. Subsequent to treatment discontinuation, survival was follow-up every 3 months, until the patient was dead or lost to follow-up.

### Outcomes

The primary endpoint was safety, graded according to CTCAE v5.0. The ORR, DCR, DoR, TTR, PFS, and OS were the secondary endpoints (**Supplement**). Exploratory endpoints analyzed the association between treatment response and pretreatment biomarkers, including TMB, gene mutation, HRD score and immune cells. Biomarker analysis was performed for patients who had sufficient tissue samples at baseline using targeted sequencing and WTS.

### DNA isolation and capture-based targeted DNA sequencing

The methods for DNA isolation and DNA sequencing were as previously described in ref. ^40^. In brief, tissue DNA was extracted from formalin-fixed, paraffin-embedded (FFPE) tumor tissues using QIAamp DNA FFPE tissue kit (Qiagen, Hilden, Germany). DNA fragments between 200 and 400 bp were then purified using Agencourt AMPure XP Kit (Beckman Coulter, CA, USA). A commercial panel consisting of 520 genes (OncoScreen Plus), spanning 1.86 megabases of the human genome was used for target capture. Indexed samples were sequenced on Nextseq 500 (Illumina, Inc., CA, USA) with paired-end reads and average sequencing depth of 1000× for tissue samples. All the procedures were performed in a commercial clinical laboratory (Burning Rock Biotech) accredited by the College of American Pathologist and certified by the Clinical Laboratory Improvement Amendments (CLIA).

### DNA sequencing data analysis

The sequencing data were first mapped to the reference human genome (hg19) using Burrows-Wheeler Aligner version 0.7.10. Local alignment optimization, duplication marking and variant calling were performed using Genome Analysis Tool Kit version 3.2, and VarScan version 2.4.3, respectively. Sequencing data of corresponding white blood cells were used to filter out germline variants and clonal haematopoiesis. Base calling in tissue samples required at least 8 supporting reads for single nucleotide variations (SNVs) and 5 supporting reads for insertion-deletion variations (Indels), respectively.

The mutation status of mTOR pathway was determined by the presence or absence of any mutation in the mTOR-related genes (Supplementary Table 7). Patients with any mutation in the mTOR-related genes were classified as mTOR pathway mutant, whereas those without mutations were classified as wild-type. TMB per patient was computed as a ratio between the total number of non-synonymous mutations detected and the total coding region size of the panel. Loss of heterozygosity (LOH), telomere allelic imbalance (TAI) and large-scale state transitions (LST) were calculated as previously described.^41^ The HRD score was calculated as the sum of LOH, TAI and LST scores.

### RNA sequencing and data analysis

The methods for RNA sequencing were as previously described in ref. ^42^. RNA was isolated from FFPE samples using an AllPrep DNA/RNA FFPE Kit (Qiagen, Hilden, Germany). Fragmented RNA was subjected to strand-specific cDNA synthesis, followed by dA-tailing, unique molecular identifier (UMI) adaptor ligation, PCR amplification, and hybridization with capture probe baits. The prepared NGS libraries were sequenced on a NovaSeq 6000 system (Illumina, Inc., San Diego, CA, USA). The immune cell scores were generated using single-sample Gene Set Enrichment Analysis (ssGSEA) of the corresponding gene set obtained from previously published literature (Supplementary Table 7).^43–45^ The upper quartile of each immune cell score was used as the threshold to classify patients into high- and low-expression groups.

### Statistical analysis

This study was exploratory and designed to detect signals for further investigation, with safety as the primary endpoint. No statistical hypothesis was made, and no formal sample size calculation was performed. Enrollment of approximately 40 patients was planned and considered sufficient to capture preliminary safety signals. Safety was assessed in all patients who received at least one dose of study drug. Efficacy was assessed in patients who had at least one post-treatment tumor evaluation. All statistical analyses were performed using R version 3.4.1. Safety outcomes were summarized with descriptive statistics. The 95% CIs for ORR and DCR were calculated using the Clopper-Pearson method. Survival outcomes were assessed by the Kaplan-Meier method. We estimated 95% CIs for survival outcomes using the Brookmeyer-Crowley method.

## Supplementary information

Supplement-clean version

Study protocol

## Data Availability

The data collected in this study are available from the corresponding author on reasonable request. Raw sequence data of this paper have been stored in the China National Center for Bioinformation (https://ngdc.cncb.ac.cn/omix, OMIX ID: OMIX008706 and OMIX008707).
