# Supplementary material for: Induction chemotherapy followed by camrelizumab plus apatinib and chemotherapy as first-line treatment for extensive-stage small-cell lung cancer: a multicenter, single-arm trial
Source: Signal Transduct Target Ther. 2025 Feb 18;10:65. doi: 10.1038/s41392-025-02153-7 (PMC11833049; doi:10.1038/s41392-025-02153-7)
Supplement: Supplementary file 1 — Supplement-clean version [file 41392_2025_2153_MOESM1_ESM.docx]

Supplementary Materials for

Induction chemotherapy followed by camrelizumab plus apatinib and chemotherapy as first-line treatment for extensive-stage small-cell lung cancer: a multicenter, single-arm trial

Ming Liu^1#^, Guihuan Qiu^1#^, Wenhui Guan^1#^, Xiaohong Xie^1^, Xinqing Lin^1^, Zhanhong Xie^1^, Jiexia Zhang^1^, Yinyin Qin^1^, Haijian Du^2^, Xin Chen^3^, Yu Deng^4^, Shiyue Li^1*^, Nanshan Zhong^1*^, Chengzhi Zhou^1*^

Correspondence to:

Prof. Nanshan Zhong, nanshan@vip.163.com

Prof. Chengzhi Zhou, [docotorzcz@163.com](mailto:docotorzcz@163.com)

Prof. Shiyue Li, lishiyue@188.com

**This PDF file includes:**

Supplementary Methods

Tables S1 to S7

**Supplementary Methods**

**Dose modifications**

Dose adjustments for camrelizumab were not permitted but could be delayed or discontinued. Dose adjustments, treatment interruptions, and discontinuations were allowed for etoposide, carboplatin, and apatinib to manage adverse events. If intolerable toxicity occurred, apatinib dose could be adjusted to 250 mg every other day (250mg qod). If intolerance persisted, apatinib should be discontinued until toxicity recovered to grade ≤1, and then apatinib 250 mg qd was continued. Permanent discontinuation of apatinib was recommended if grade ≥3 toxicity recured. If toxicity from etoposide and/or carboplatin did not recovery within the same treatment cycle, administration could be delayed for no longer than 9 weeks. After 2 dose reductions or treatment delays exceeding 9 weeks due to toxicity, treatment with etoposide and/or carboplatin was discontinued unless the investigator believed that patients could benefit from continued treatment.

**Definitions of the endpoints**

Objective response rate was determined as the percentage of patients who had complete response (CR) or partial response (PR) as best overall response per RECIST v1.1. Disease control rate was defined as the percentage of patients with CR, PR or stable disease. Duration of response was determined as the interval from the initial documented objective response to disease progression or death from any cause. Time to response was determined as the interval from first dose to initial documented objective response. Progression-free survival was determined as the interval from initiation of the study dose to disease progression or death, whichever occurred first. Overall survival was determined as the interval from initiation of the study dose to death from any cause.

**Table S1.** Vascular invasion sites and grade

|  | Patients (n=40) |
| --- | --- |
| Superior Vena Cava |  |
| Grade 1 | 25 (62.5) |
| Grade 3 | 10 (25.0) |
| Main Pulmonary Artery |  |
| Grade 1 | 14 (35.0) |
| Grade 3 | 21 (52.5) |
| Main Pulmonary Vein |  |
| Grade 1 | 13 (32.5) |
| Grade 2 | 1 (2.5) |
| Grade 3 | 21 (52.5) |
| Aorta |  |
| Grade 1 | 35 (87.5) |
| Lobar Pulmonary Artery |  |
| Grade 1 | 3 (7.5) |
| Grade 3 | 2 (5.0) |
| No vascular invasion | 29 (72.5) |

Data are n (%).

**Table S2.** Treatment cycles during the whole treatment phase

|  | Patients (n = 36) |
| --- | --- |
| Median EC cycles | 6 (2-6) |
| EC more than 4 cycles | 35 (97.2%) |
| 6 cycles of EC | 30 (83.3%) |
| Median treatment cycles of camrelizumab | 7 (2-35) |
| Median treatment duration of apatinib, months | 5.23 (95%CI: 4.73-5.73) |
| Treatment cycles of four-drug combination therapy |  |
| 2 cycles | 6/35 (17.1%) |
| 3 cycles | 5/35 (14.3%) |
| 4 cycles | 24/35 (68.6%) |
| Treatment more than 12 months | 6 (16.67%) |

EC: etoposide and carboplatin.

**Table S3.** Reasons for not completing 4 cycles of four-drug combination therapy

| Reason | Treatment cycles (n = 35) | |
| --- | --- | --- |
|  | 2 cycles | 3 cycles |
| Adverse events | 4 (11.4) | 3 (8.6) |
| Withdrew of informed consent | 1 (2.9) | 0 |
| Physician decision | 1 (2.9) | 0 |
| Omission of prescription | 0 | 1 (2.9) |
| Death, unrelated to treatment | 0 | 1 (2.9) |

Data are n (%).

**Table S4.** Camrelizumab-related adverse events (n = 35)

|  | Total | Grade 3 and above |
| --- | --- | --- |
| Hyperthyroidism | 13 (37.1) | 0 |
| Increased alanine aminotransferase | 8 (22.9) | 5 (14.3) |
| Increased thyroid stimulating hormone | 7 (20.0) | 0 |
| Diarrhea | 7 (20.0) | 0 |
| Increased aspartate aminotransferase | 7 (20.0) | 3 (8.6) |
| Immune-mediated hepatitis | 4 (11.4) | 1 (2.9) |
| Leukopenia | 3 (8.6) | 0 |
| Hypothyroidism | 3 (8.6) | 0 |
| Immune-mediated pneumonitis | 3 (8.6) | 2 (5.7) |
| Anemia (Lower hemoglobin) | 3 (8.6) | 0 |
| Bloating | 2 (5.7) | 0 |
| Hyperglycemia | 2 (5.7) | 2 (5.7) |
| Infusion-related reactions (itchy skin) | 2 (5.7) | 0 |
| Thrombocytopenia | 2 (5.7) | 0 |
| Neutropenia | 2 (5.7) | 1 (2.9) |
| Capillary hyperplasia | 2 (5.7) | 0 |

Data are n (%).

**Table S5.** Apatinib-related adverse events (n = 35)

|  | Total | Grade 3 and above |
| --- | --- | --- |
| Hand foot syndrome | 13 (37.1) | 1 (2.9) |
| Leukopenia | 9 (25.7) | 0 |
| Neutropenia | 9 (25.7) | 4 (11.4) |
| Anemia (Lower hemoglobin) | 8 (22.9) | 1 (2.9) |
| Increased aspartate aminotransferase | 7 (20.0) | 3 (8.6) |
| Increased alanine aminotransferase | 6 (17.1) | 4 (11.4) |
| Hemoptysis | 6 (17.1) | 0 |
| Hypokalemia | 5 (14.3) | 0 |
| Hyponatremia | 5 (14.3) | 4 (11.4) |
| Hypertension | 5 (14.3) | 4 (11.4) |
| Thrombocytopenia | 4 (11.4) | 0 |
| Increased r-glutaminase | 3 (8.6) | 3 (8.6) |
| Sore throat | 3 (8.6) | 0 |
| Focal edema (Sole of foot) | 2 (5.7) | 0 |
| Oral mucositis | 2 (5.7) | 0 |

Data are n (%).

**Table S6.** Antitumor response in all enrolled patients

|  | Patients  (n = 40) |
| --- | --- |
| Response to induction treatment |  |
| ORR | 24 (60.0, 43.3-75.1) |
| DCR | 35 (87.5, 73.2-95.8) |
| Overall response |  |
| ORR | 32 (80.0, 64.4-90.9) |
| Confirmed ORR | 28 (70.0, 53.5-83.4) |
| DCR | 35 (87.5, 73.2-95.8) |
| Best overall response |  |
| Partial response | 32 (80.0, 64.4-90.9) |
| Stable disease | 3 (7.5, 15.7-20.4) |
| Progressive disease | 1 (2.5, 0.6-13.2) |
| Unknown | 4 (10.0, 2.8-23.7) |

Data are n (%) or n (%, 95% CI). ORR, objective response rate; DCR, disease control rate.

**Table S7.** Signature-related genes in exploratory biomarker analysis

| Signature | Gene | Data type |
| --- | --- | --- |
| mTOR pathway | *RICTOR, RPTOR, TSC1, TSC2, RHEB, MTOR* | Mutation |
| NK cells | *KIR2DL1, KIR2DL2, KIR2DL3, KIR2DL4, KIR2DL5A, KIR2DS1, KIR2DS2, KIR2DS3, KIR2DS5, KIR3DL1, KIR3DL2, KIR3DL3, KLRC2, KLRC3, KLRC4, KLRD1, PRF1, SAMD3, SH2D1B, TBX21* | Expression |
| Interferons | *IFNA10, IFNA13, IFNA14, IFNA16, IFNA17, IFNA2, IFNA21, IFNA4, IFNA5, IFNA6, IFNA7, IFNA8, IFNB1, IFNE, IFNG, IFNK, IFNW1* | Expression |
| Cancer-associated fibroblasts | *COL1A1, COL1A2, COL5A1, ACTA2, FGF2, FAP, LRP1, CD248, COL6A1, COL6A2, COL6A3, CXCL12, FBLN1, LUM, MFAP5, MMP3, MMP2, PDGFRB, PDGFRA* | Expression |
