# Supplementary material for: Induction chemotherapy followed by camrelizumab plus apatinib and chemotherapy as first-line treatment for extensive-stage small-cell lung cancer: a multicenter, single-arm trial
Source: Signal Transduct Target Ther. 2025 Feb 18;10:65. doi: 10.1038/s41392-025-02153-7 (PMC11833049; doi:10.1038/s41392-025-02153-7)
Supplement: Supplementary file 2 — Study protocol [file 41392_2025_2153_MOESM2_ESM.docx]

**Induction Chemotherapy Followed by** **Camrelizumab, Apatinib plus Chemotherapy as First-line Treatment for Extensive-stage Small-cell Lung Cancer: A Multicenter Single-arm Study with Biomarker Analysis**

**Clinical study protocol**

| Version No. | 1.2 |
| --- | --- |
| Date | 03 September,2021 |
| The primary clinical research center | The First Affiliated Hospital of Guangzhou Medical University, Guangzhou, China |

**Content**

[ABSTRACT 1](#_Toc170253663)

[SCHEDULE OF ACTIVITIES 8](#_Toc170253664)

[1 BACKGROUND OF THE STUDY 10](#_Toc170253665)

[2 STUDY ENPOINTS 13](#_Toc170253666)

[2.1 Primary Endpoints 13](#_Toc170253667)

[2.2 Secondary Endpoints 13](#_Toc170253668)

[3 STUDY DESIGN 13](#_Toc170253669)

[3.1 Schematic Design 13](#_Toc170253670)

[4 SAMPLE SIZE 14](#_Toc170253671)

[5 SUBJECT SELETION 14](#_Toc170253672)

[5.1 Inclusion Criteria 14](#_Toc170253673)

[5.2 Exclusion Criteria 14](#_Toc170253674)

[6 WITHDRAWAL CRITERIA 16](#_Toc170253675)

[7 TERMINATION CRITERIA 16](#_Toc170253676)

[8 TRETAMENT REGIMEN AND DOSAGE 16](#_Toc170253677)

[9 DOSE AJUSTMENT AND DISCONTINUATION OF ADMINISTRATION 17](#_Toc170253678)

[9.1 General Rules for Dose Adjustment 17](#_Toc170253679)

[9.2 Camrelizumab Dose Adjustment 17](#_Toc170253680)

[9.3 Apatinib Dose Adjustment 19](#_Toc170253681)

[9.4 Chemotherapy Dose Adjustment 20](#_Toc170253682)

[10 CONCOMITANT THERAPY 21](#_Toc170253683)

[10.1 Hematologic Support 21](#_Toc170253684)

[10.2 Treatment of Symptoms of Non-Hematologic Toxicity 21](#_Toc170253685)

[10.3 Other Anti-tumor/Cancer or Investigational Drugs 22](#_Toc170253686)

[11 STUDY PROCEDURES 22](#_Toc170253687)

[11.1 Screening Period 23](#_Toc170253688)

[11.2 Treatment period 24](#_Toc170253689)

[11.3 End of Treatment 25](#_Toc170253690)

[11.4 Safety Follow-Up 26](#_Toc170253691)

[12 CLINICAL EVALUATION 27](#_Toc170253692)

[12.1 Primary Efficacy Indicators 27](#_Toc170253693)

[12.2 Secondary Efficacy Indicators 27](#_Toc170253694)

[12.3 Imaging Scan Site 27](#_Toc170253695)

[12.4 Evaluation Time Point 27](#_Toc170253696)

[13 SECURITY ASSESSMENT 28](#_Toc170253697)

[13.1 Security Indicators 28](#_Toc170253698)

[13.1.1 Indicators for routine laboratory safety assessment 28](#_Toc170253699)

[13.1.2 Physical examination 28](#_Toc170253700)

[13.2 Adverse Event 30](#_Toc170253701)

[13.3 AE Classification 30](#_Toc170253702)

[13.4 AE Records 31](#_Toc170253703)

[13.5 AE Causality Assessment with Investigational Medicinal Product 31](#_Toc170253704)

[13.6 Serious Adverse Events SAE 31](#_Toc170253705)

[13.7 SAE's Reporting Procedures 32](#_Toc170253706)

[14 DATA COLLECTION AND DATA MANAGEMENT 33](#_Toc170253707)

[14.1 Data Auditing and Monitoring 33](#_Toc170253708)

[14.2 Database lockout 33](#_Toc170253709)

[14.3 Statistical Analysis of Data Sets 33](#_Toc170253710)

[14.4 Statistical analysis plan 33](#_Toc170253711)

[15 MEDICINES MANAGEMENT 34](#_Toc170253712)

[16 ETHICAL CONSIDERATIONS 34](#_Toc170253713)

[17 STUDY PLAN 35](#_Toc170253714)

**Table of Content**

[**Table 1. Dose Adjustment Provisions for Camrelizumab** 18](#_Toc170253762)

[**Table 2. Treatment Recommendations for Camrelizumab Infusion Reactions** 18](#_Toc170253763)

[**Table 3. Dose Adjustment Provisions for Apatinib** 19](#_Toc170253764)

[**Table 4. Chemotherapy Dose Modification due to Hematologic Toxicity** 20](#_Toc170253765)

[**Table 5. Dose Modification or Treatment Discontinuation due to Non-Hematologic Toxicity** 21](#_Toc170253766)

[**Table 6. Dose Modifications of Etoposide for Subjects with Renal Impairment.** 21](#_Toc170253767)

[**Table 7. Screening Visits Process** 23](#_Toc170253768)

[**Table 8. Treatment Visits Process** 24](#_Toc170253769)

[**Table 9. End-of-Treatment Visits Process** 25](#_Toc170253770)

[**Table 10. Safety Follow-up Process** 26](#_Toc170253771)

[**Table 11. Routine Laboratory Safety Assessment** 28](#_Toc170253772)

[**Table 12. Criteria for Determining the Relationship between AE and Medications** 31](#_Toc170253773)

# ABSTRACT

| **Study Title** | Induction Chemotherapy Followed by Camrelizumab, Apatinib plus Chemotherapy as First-line Treatment for Extensive-stage Small-cell Lung Cancer: A Multicenter Single-arm Study with Biomarker Analysis |
| --- | --- |
| **Version No.** | 1.2 |
| **The Primary Clinical Research Center** | The First Affiliated Hospital of Guangzhou Medical University, Guangzhou, China |
| **The Primary Investigator** | Professor Chengzhi Zhou |
| **Participating Study Sites** | Zhujiang Hospital affiliated to Southern Medical University |
| **Trial Design** | Prospective, phase II clinical trial |
| **Participants** | Patients with extensive-stage small cell lung cancer who did not receive systemic treatment |
| **Study Objectives** | To evaluate the safety and efficacy of chemotherapy combined with PD-1 antibody camrelizumab and apatinib in the first-line treatment of extensive stage small cell lung. |
| **Study Endpoints** | **Key endpoints**   - Safety (per CTCAE 5.0 criteria)   **Secondary endpoints**   - Disease control rate (DCR) - Duration of response (DoR) - Objective response rate(ORR per RECIST v1.1) - Progression-Free Survival (PFS per RECIST v1.1) - Overall surviral(OS)   **Exploratory endpoint**   - Exploratory analysis（tumor mutation burden (TMB), gene mutation, homologous recombination deficiency (HRD) score and immune cells） |
| **Study Drug** | Camrelizumab、apatinib、Etoposide、Carboplatin |
| **Inclusion Criteria** | 1. Pathologically confirmed extensive stage small cell lung cancer; 2. Extensive small cell lung cancer (per the Veterans Administration 3. Lung Study Group (VALG) staging system) not receiving systemic treatment; 4. Received prior surgery and adjuvant therapy for curative intent such as radiotherapy and chemotherapy; at least 6 months of treatment-free period since last chemotherapy, radiotherapy, or chemoradiotherapy to diagnosis of extensive-stage SCLC; 5. Measurable lesions according to Response Evaluation Criteria in Solid Tumors (RECIST) version 1.1; 6. Male or female ≥ 18 years of age and ≤ 75 years of age; 7. ECOG performance status score of 0-1; 8. Life expectancy ≥ 12 weeks; 9. Adequate organ system function (excluding the use of any blood components and cell growth factors during screening):    1. Hemoglobin ≥ 9 g/dL    2. Absolute neutrophil count ≥1.5×109/L    3. Platelets ≥100 × 109/L    4. Serum albumin ≥ 3/dL    5. Total bilirubin ≤ ULN    6. ALT and AST ≤ 1.5 × ULN; AKP ≤ 2.5 × ULN    7. Serum creatinine ≤1.5 ULN or creatinine clearance(CrCl) ≥60 mL/min (Cockcroft -Gault formula). Negative urine protein or 24-hour urine protein quantification <1.0 g    8. Thyroid stimulating hormone (TSH) ≤ULN (subjects with normal T3 and T4 levels are allowed to be enrolled although TSH＞ULN) 10. Contraception (e.g., IUD, contraceptives, or condoms); a negative pregnancy test within 7 days prior to enrollment for women of childbearing age; 11. Subjects voluntarily enrolled in this study and signed an informed consent form. |
| **Exclusion Criteria** | 1. Prior treatment with apatinib, anti-programmed cell death (PD)-1, anti-PD-1, or other immunotherapy against PD-1/PD- L1; 2. Carcinomatous meningitis, subjects with asymptomatic brain metastases and did not require immediate radiotherapy could be included in the study; 3. Other malignancies diagnosed and/or treated within 5 years prior to enrollment, except cured basal cell carcinoma of the skin and carcinoma in situ of the uterine cervix; 4. There are a variety of factors that affect the oral administration of medications, such as inability to swallow, posterior gastrointestinal resection, chronic diarrhea, intestinal obstruction, etc.; 5. Have an uncontrollable pleural effusion, pericardial effusion, or ascites that cannot be controlled by drainage or other procedures; 6. Subjects with spinal cord compression that has not been cured or resolved by surgery and/or radiotherapy, or patients with spinal cord compression diagnosed after treatment, who do not have clinical evidence of disease stabilization ≥1 week prior to enrollment; 7. Subjects with hypertension that is not well controlled with oral antihypertensive medication (systolic blood pressure > 140 mmHg, diastolic blood pressure >90 mmHg), with Grade I or greater myocardial ischemia or myocardial infarction, Grade I or greater arrhythmia (including QT interval ≥440 ms) or cardiac insufficiency; 8. Subjects with signs of bleeding or predisposition to bleeding, regardless of the degree of bleeding, within 2 months prior to the first dose; history of hemoptysis (defined as more than 2.5 ml of bright red blood) or unhealed wounds, ulcers, or fractures in the previous 2 weeks; 9. Incomplete recovery from adverse events due to prior therapy, except for alopecia which did not recover to grade ≤1; 10. Major surgery or significant traumatic injury within 28 days prior to enrollment; 11. An arterial or venous thromboembolic event within 6 months, such as transient ischemic attack, deep vein thrombosis, pulmonary embolism, and other cerebrovascular accidents; 12. Those with a history of substance abuse, inability to quit, or a mental disorder; 13. Suffering from a serious and/or uncontrollable illness; 14. Vaccination or attenuated vaccine within 4 weeks prior to the first injection; 15. Known allergy to the study drugs or excipients, history of severe allergic reaction to any monoclonal antibody; history of allergy to carboplatin or etoposide ; 16. Active autoimmune disease requiring systemic therapy within 2 years prior to first dose; 17. Immunosuppressive, systemic or local absorbable hormones (prednisone or other therapeutic hormones at doses > 10 mg/day) were administered to the patient. Immunosuppressive therapy was administered and continued for 2 weeks after the first dose; 18. Participation in a clinical trial of another anticancer drug within 4 weeks; 19. In the judgment of the investigator, there are other factors that may have led to the termination of the study. |
| **Withdrawal Criteria** | - Subjects withdrew informed consent. - Progression of the disease on imaging. - Those who cannot tolerate toxicity. - Subjects missed visits or had positive blood HCG. - Other situations where the investigator felt it necessary to withdraw from the study. |
| **Termination Criteria** | - Unintended or unacceptable risks to the subject are identified. - The study drug/trial treatment is ineffective, or it is pointless to continue the trial. - For reasons such as difficulties in subject enrollment or frequent protocol deviations, the investigator decided to terminate the study. |
| **Treatment Regimen and Dosage** | 1. **Induction treatment stage: chemotherapy induction 2 cycles,** **q3w**  - Etoposide: 100 mg/m2 IV Day 1-3; - Carboplatin: AUC 5 mg/mL/min IV Day 1  1. **Combination treatment stage: chemotherapy + immunotherapy + antivascular therapy 2-4 cycles, q3w**  - Etoposide: 100 mg/m2 IV Day 1-3 - Carboplatin: AUC 5 mg/mL/min IV Day 1 - Camrelizumab: 200 mg IV Day 1 - Apatinib: 250 mg PO QD  1. **Maintenance treatment stage: immunotherapy + antivascular therapy, q3w**  - Camrelizumab: 200mg IV Day 1 - Apatinib: 250mg PO QD |
| **Study Plan** | 2020.9 - 2021.1 Submissions, Ethical Review of Research Units.  2021.2-2022.2 The patients were enrolled and the data were recorded.  2022.3-2023.03 Complete follow-up and data collection of all enrolled patients.  2023.04-2024.12 Data analysis, paper writing and contributions. |

# SCHEDULE OF ACTIVITIES

| **Stage** | **Screening Period** | | **Treatment Period**  **(21 days/cycle)** | | **End of Treatment**  D1±3 | **Safety Follow-up**  (30 days) | **Survival Follow-up**  Every 3 months |  |
| --- | --- | --- | --- | --- | --- | --- | --- | --- |
|  | -28-1 days | -7-1 days | Cycle 1  D1±3 | Follow-up cycle  D1±3 |  |  |  |  |
| Baseline | | | |  |  |  | |  |
| Written Informed Consent Form | X |  |  |  |  |  |  |  |
| Inclusion/Exclusion Criteria | X |  |  |  |  |  |  |  |
| Demographics/Medical History/Previous Medication | X |  |  |  |  |  |  |  |
| Vital Signs | X |  |  | X | X | X |  |  |
| Weight, height | X |  |  | X | X | X |  |  |
| Physical Examination | X |  |  | X | X | X |  |  |
| ECOG PS Sore | X |  |  | X | X | X |  |  |
| Laboratory Tests | | | |  |  |  | |  |
| 12-lead ECG | X |  | X | X | X | X |  |  |
| Hematology/Blood Biochemistry Urinalysis/Urine  Routine/Stool Tests |  | X |  | X | X | X |  |  |
| Coagulation Function |  | X |  | X | X | X |  |  |
| Pregnancy Test |  | X |  |  | X |  |  |  |
| Thyroid Function | X |  |  | X | X |  |  |  |
| Myocardial Enzyme Profile |  | X |  | Add when clinically indicated | | |  |  |
| Pituitary Function Tests | X |  |  | Add when clinically indicated | | |  |  |
| HIV,, , HBV and HCV Tests | X |  |  |  |  |  |  |  |
| Echocardiography |  | X |  | Add when clinically indicated | | |  |  |
| Pulmonary Function Tests and Arterial Blood Gas (ABG) Analysis | X |  |  | Add when clinically indicated | | |  |  |
| Medication |  |  | 1-2 cycles of induction treatment chemotherapy, 3-4 cycles of combination treatment , and follow by maitanence treatment. | |  | |  |  |
| AE Assessment | X |  |  | X | X | X |  |  |
| Concurrent Medications | X |  | X | X | X | X |  |  |
| After-Treatment Visits | | | |  |  |  | |  |
| Survival Follow-up |  |  |  |  |  | X | X |  |
| Anti-Tumor Therapy |  |  |  |  |  | X | X |  |
| Evaluation of Efficacy | | | |  |  |  | |  |
| Imaging Evaluation | X |  | Assess on day 21 of cycle 1, and every 6 weeks thereafter until disease progression or the start of a new treatment. | | X |  |  | |

# BACKGROUND OF THE STUDY

SCLC accounts for about 15% of all lung cancers, and it can be divided into two categories: limited stage (LS-SCLC) and extensive stage (ES-SCLC)^1^ . Limited stage SCLC is confined to one side of the lung and may be present in lymph nodes on the same side of the chest. If the cancer spreads to the other side of the lung, to lymph nodes on the other side of the chest, or to distant organs, it is characterize as extensive stage. Few patients diagnosed with extensive or unresectable limited stage are cured with surgery. Regardless of the stage, systemic platinum-based chemotherapy is the mainstay of treatment for SCLC. Although SCLC is sensitive to platinum-based chemotherapy, with patients achieving remission rates of 50-90%, recurrence and rapid progression of the disease are very common^2^ .

After close to 20 years of using platinum-based chemotherapy as the standard of care, immune checkpoint inhibitors are finally making a breakthrough in SCLC treatment. Bristol-Myers Squibb's PD-1 inhibitor Opdivo (Nivolumab) received accelerated approval in August 2018 as a third-line therapy for patients with ES-SCLC. This was followed in June 2019 by Merck Sharp & Dohme's Keytruda (Pembrolizumab) was also approved for a third-line indication; two PD-L1 antibodies, Roche's Tecentriq (Atezolizumab) in March 2019 and AstraZeneca's Imfinzi (Durvalumab) in March 2020, were even more consecutively approved as first-line treatments in combination with chemotherapy for patients with ES-SCLC^3^ .

In addition, Yervoy (ipilimumab), a CTLA-4 inhibitor developed by BMS, is not FDA-approved for the treatment of SCLC, but it is included in the National Comprehensive Cancer Network (NCCN) guidelines in combination with Opdivo as a second-line therapy for the treatment of patients with SCLC who have experienced disease recurrence after first-line chemotherapy^4^ .

The two immune checkpoint inhibitors currently approved for broad-stage small cell lung cancer indications are both PD-L1 antibodies. 2020 ASCO reported updated data on two PD-1 antibodies, Nivolumab and Pembrolizumab, in first-line combination chemotherapy. The primary endpoint of PFS was achieved with Nivolumab in combination with CE versus CE chemotherapy, with a median PFS of 5.5 months versus 4.7 months, HR=0.68, p=0.047, and a statistically significant difference of 0.8-month. Unfortunately, the secondary endpoint of OS was not achieved in the modified study, with a median OS of 11.3 months versus 9.3 months ,p=0.14, which was not statistically significant. The Keynote-604 study also did not provide a positive OS result for first-line combination chemotherapy with pembrolizumab, although the median OS was 10.8 months versus 9.7 months, with a P-value of 0.0164, which did not meet the efficacy cutoff of 0.0128, and was therefore not statistically different. statistical difference^5^ .

The efficacy of PD-1 combination chemotherapy in the first-line treatment of small cell lung cancer is not satisfactory, whether immunization combined with other modalities, such as anti-angiogenic drugs, can further improve the efficacy? Impower-150 study^6^ gives a hint, the study set up three groups, the ABCP group (immunotherapy + anti-angiogenesis + chemotherapy), the ACP group (immunotherapy + chemotherapy), and the BCP group (anti-angiogenesis + chemotherapy). This study used platinum doublet chemotherapy plus bevacizumab, which has a well-established gold standard status for advanced non-squamous non-small cell lung cancer (NSCLC), as the control group (while other related studies used standard platinum doublet regimens as control). It was the first study to demonstrate that the addition of atezolizumab to the combination of bevacizumab, paclitaxel, and carboplatin was superior to the three-drug combination without immunotherapy, specifically showing a benefit for patients (ABCP group vs BCP group: Overall Survival (OS) 19.5 months vs 14.7 months). This represents the first and currently the only phase III clinical trial demonstrating statistically and clinically significant improvements in Progression-Free Survival (PFS) and OS with first-line immunotherapy combined with anti-angiogenesis for metastatic non-squamous NSCLC. There were also numerical improvements in Objective Response Rate (ORR) and Duration of Response (DOR) with the four-drug combination compared to the three-drug regimens, with ORR reaching 56% in the four-drug group compared to 40-41% in the three-drug groups, and DOR increasing from 6.0-8.3 months to 11.5 months. The incidence of new brain metastases in the ABCP and BCP groups was also lower than in the ACP group, at 7%, 6%, and 11.9%, respectively.The study results suggest that, based on the selection of appropriate patient populations, adding anti-angiogenic drugs to a chemotherapy + immunotherapy regimen may further enhance efficacy. Specifically, the ABC group (immunotherapy + anti-angiogenic + chemotherapy) in the Impower-150 study demonstrated better overall survival (OS 19.5 months vs. 14.7 months) compared to other combinations, providing a positive signal for first-line treatment of small-cell lung cancer.

Additionally, the Keynote-021 cohort study^7^ reached similar conclusions. This study evaluated the effectiveness of the combination therapy "chemotherapy + pembrolizumab + bevacizumab." Different combinations exhibited varying objective response rates (ORR), with the combination including bevacizumab showing a higher ORR (Group A 40%, Group B 56%, Group C 75%). This further supports the notion that adding anti-angiogenic drugs to immunotherapy may enhance treatment efficacy.

Furthermore, the PASSION study^8^ of camrelizumab focused on the combination therapy with apatinib for extensive-stage small-cell lung cancer. Preliminary results indicated an ORR of 34% in the overall patient population, including both chemotherapy-sensitive and chemotherapy-resistant patients, with a duration of response (DOR) of 6.2 months, progression-free survival (PFS) of 3.6 months, and overall survival (OS) of 8.4 months. This study suggests that the combination of camrelizumab and apatinib demonstrates favorable efficacy, especially in patients with small-cell lung cancer after platinum-based chemotherapy, and its safety profile is acceptable.

In addition, approximately 75%-90% of small cell lung cancer (SCLC) is centrally located, occurring within or up to one segment of the bronchi. Although the tumor originates from the bronchial mucosa, it tends to grow along the submucosa and infiltrate the surrounding interstitium along its longitudinal axis. SCLC exhibits characteristic lymphatic spread, with prominent involvement of lymph nodes. It often compresses, embeds, and invades structures in the lung hilum and mediastinum, such as the pericardium, major blood vessels, trachea, bronchi, esophagus, and nerves.

A retrospective analysis^9-10^ of 50 cases of SCLC examined the vascular involvement, categorizing it into three levels based on the degree of invasion. The results showed that out of the 42 cases with vascular involvement, there were a total of 84 affected vessels, including 37 instances of involvement of the pulmonary artery, 29 involving the pulmonary veins, 16 involving the superior vena cava, and 2 involving the aorta. Based on the degree of vascular involvement, 12 cases were classified as level 1, 3 cases as level 2, and 69 cases as level 3. Vascular involvement in SCLC, particularly in major blood vessels, may increase the risk of bleeding during anti-vascular therapy. It prompts consideration of whether interventions can be employed to reduce the extent of tumor invasion into the vessel walls, thereby enhancing the safety and efficacy of anti-vascular treatment for this population.

Small cell lung cancer is a highly malignant tumor sensitive to chemotherapy. Over the past few decades, platinum-based chemotherapy combined with etoposide has been widely accepted as the standard first-line treatment for SCLC. Objective response rates of around 60% have been achieved with this regimen, leading to tumor shrinkage in most patients. However, the development of resistance and subsequent relapse and metastasis commonly occur shortly after chemotherapy. Consequently, there is a need to explore an investigational clinical study focusing on the safety and efficacy of combining immune checkpoint inhibitors and anti-vascular agents with first-line chemotherapy after induction therapy for SCLC, aiming to gain preliminary insights into the potential benefits and safety of this combined approach.

**bibliography**

1. Lauren Averett Byers, Charles M Rudin. Small cell lung cancer: where do we go from here? Cancer. 2015 Mar 1;121(5):664-72.
2. Erica B Bernhardt, Shadia I Jalal. Small Cell Lung Cancer. Cancer Treat Res. 2016;170:301-22.
3. Shuhang Wang, Stefan Zimmermann, et al. Current Diagnosis and Management of Small-Cell Lung Cancer. Mayo Clin Proc. 2019 Aug;94(8):1599-1622.
4. NCCN SCLC guidelines, 2019, V3.
5. Giovanna Esposito, et al. Immunotherapy in Small Cell Lung Cancer. cancers (Basel). 2020 Sep 4;12(9):E2522.
6. Martin Reck, et al. Atezolizumab plus bevacizumab and chemotherapy in non-small-cell lung cancer (IMpower150): key subgroup analyses of patients with EGFR mutations or baseline liver metastases in a randomized, open-label phase 3 trial. Lancet Respir Med. 2019 May;7(5):387- 401.
7. Pembrolizumab and platinum-based chemotherapy as first-line therapy for advanced non-small-cell lung cancer: Phase 1 cohorts from the KEYNOTE-021 study. Lung Cancer 125(2018)273-281.
8. Fan Y, et al. Camrelizumab Plus Apatinib in Extensive-Stage SCLC (PASSION): A Multicenter, Two-Stage, Phase 2 Trial. J Thorac Oncol. 2021 Feb;16(2):299-309.
9. Nobukata Kazawa, et al. Small cell lung carcinoma: Eight types of extension and spread on computed tomography. Journal of Computer Assisted Tomography. Journal of Computer Assisted Tomography. 2006;30(4): 653-661.
10. V Raptopoulos, et al. The use of helical CT and CT angiography to predict vascular involvement from pancreatic cancer: correlation with findings at surgery. AJR Am J Roentgenol. 1997 Apr;168(4):971-7.

# STUDY ENPOINTS

## Primary Endpoints

- Safety (per CTCAE 5.0 criteria)

## Secondary Endpoints

- Disease control rate (DCR)
- Duration of response (DoR)
- Objective response rate(ORR per RECIST v1.1)
- Progression-Free Survival (PFS per RECIST v1.1)
- Overall surviral(OS))

# STUDY DESIGN

## Schematic Design

This study is a prospective, multi-cohort clinical trial designed to evaluate the safety and efficacy of chemotherapy(Etoposide + Carboplatin) in combination with the PD-1 antibody camrelizumab and the anti-angiogenic drug apatinib in the treatment of patients with extensive stage small cell lung cancer.

Study objectives: To explore the safety of induction chemotherapy followed by chemotherapy combined with immune and anti-angiogenic drugs for the first-line treatment of patients with ES-SCLC; (2) To explore the efficacy of induction chemotherapy followed by chemotherapy combined with immune and anti-angiogenic drugs for the first-line treatment of patients with ES-SCLC; (3) To explore the precision treatment population of patients with extensive small cell lung cancer treated with chemotherapy combined with immunological and anti-angiogenic agents after induction chemotherapy.

If subjects did not withdraw consent from the trial or were deemed by the investigator to be unsuitable for further trials, and each subject will be treated until disease progression or, drug-induced toxicities are intolerable, followed by a survival follow-up period.


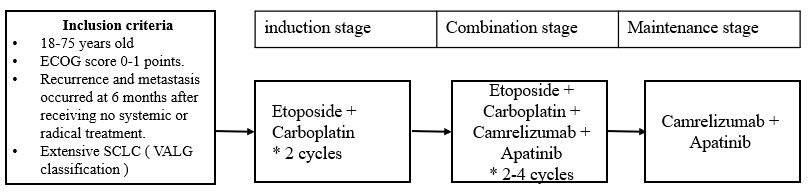
(1) Etoposide: 100 mg/m2 IV Day 1-3; Carboplatin: AUC 5 mg/mL/min IV Day 1; Camrelizumab: 200 mg IV Day 1; Apatinib: 250 mg Po QD.

**Figure 1. Study Flow Chart**

# SAMPLE SIZE

The study plans to enroll 36 subjects.

# SUBJECT SELETION

## Inclusion Criteria

1. Pathologically confirmed extensive stage small cell lung cancer;
2. Extensive small cell lung cancer (per the Veterans Administration
3. Lung Study Group (VALG) staging system) not receiving systemic treatment;
4. Received prior surgery and adjuvant therapy for curative intent such as radiotherapy and chemotherapy; at least 6 months of treatment-free period since last chemotherapy, radiotherapy, or chemoradiotherapy to diagnosis of extensive-stage SCLC;
5. Measurable lesions according to Response Evaluation Criteria in Solid Tumors (RECIST) version 1.1;
6. Male or female ≥ 18 years of age and ≤ 75 years of age;
7. ECOG performance status score of 0-1;
8. Life expectancy ≥ 12 weeks;
9. Adequate organ system function (excluding the use of any blood components and cell growth factors during screening):
   1. Hemoglobin ≥ 9 g/dL
   2. Absolute neutrophil count ≥1.5×109/L
   3. Platelets ≥100 × 109/L
   4. Serum albumin ≥ 3/dL
   5. Total bilirubin ≤ ULN
   6. ALT and AST ≤ 1.5 × ULN; AKP ≤ 2.5 × ULN
   7. Serum creatinine ≤1.5 ULN or creatinine clearance(CrCl) ≥60 mL/min (Cockcroft -Gault formula). Negative urine protein or 24-hour urine protein quantification <1.0 g
   8. Thyroid stimulating hormone (TSH) ≤ULN (subjects with normal T3 and T4 levels are allowed to be enrolled although TSH＞ULN)
10. Contraception (e.g., IUD, contraceptives, or condoms); a negative pregnancy test within 7 days prior to enrollment for women of childbearing age;
11. Subjects voluntarily enrolled in this study and signed an informed consent form.

## Exclusion Criteria

1. Prior treatment with apatinib, anti-programmed cell death (PD)-1, anti-PD-1, or other immunotherapy against PD-1/PD- L1;
2. Carcinomatous meningitis, subjects with asymptomatic brain metastases and did not require immediate radiotherapy could be included in the study;
3. Other malignancies diagnosed and/or treated within 5 years prior to enrollment, except cured basal cell carcinoma of the skin and carcinoma in situ of the uterine cervix;
4. There are a variety of factors that affect the oral administration of medications, such as inability to swallow, posterior gastrointestinal resection, chronic diarrhea, intestinal obstruction, etc.;
5. Have an uncontrollable pleural effusion, pericardial effusion, or ascites that cannot be controlled by drainage or other procedures;
6. Subjects with spinal cord compression that has not been cured or resolved by surgery and/or radiotherapy, or patients with spinal cord compression diagnosed after treatment, who do not have clinical evidence of disease stabilization ≥1 week prior to enrollment;
7. Subjects with hypertension that is not well controlled with oral antihypertensive medication (systolic blood pressure > 140 mmHg, diastolic blood pressure >90 mmHg), with Grade I or greater myocardial ischemia or myocardial infarction, Grade I or greater arrhythmia (including QT interval ≥440 ms) or cardiac insufficiency;
8. Subjects with signs of bleeding or predisposition to bleeding, regardless of the degree of bleeding, within 2 months prior to the first dose; history of hemoptysis (defined as more than 2.5 ml of bright red blood) or unhealed wounds, ulcers, or fractures in the previous 2 weeks;
9. Incomplete recovery from adverse events due to prior therapy, except for alopecia which did not recover to grade ≤1;
10. Major surgery or significant traumatic injury within 28 days prior to enrollment;
11. An arterial or venous thromboembolic event within 6 months, such as transient ischemic attack, deep vein thrombosis, pulmonary embolism, and other cerebrovascular accidents;
12. Those with a history of substance abuse, inability to quit, or a mental disorder;
13. Suffering from a serious and/or uncontrollable illness;
14. Vaccination or attenuated vaccine within 4 weeks prior to the first injection;
15. Known allergy to the study drugs or excipients, history of severe allergic reaction to any monoclonal antibody; history of allergy to carboplatin or etoposide ;
16. Active autoimmune disease requiring systemic therapy within 2 years prior to first dose;
17. Immunosuppressive, systemic or local absorbable hormones (prednisone or other therapeutic hormones at doses > 10 mg/day) were administered to the patient. Immunosuppressive therapy was administered and continued for 2 weeks after the first dose;
18. Participation in a clinical trial of another anticancer drug within 4 weeks;
19. In the judgment of the investigator, there are other factors that may have led to the termination of the study.

# WITHDRAWAL CRITERIA

- Subjects withdrew informed consent.
- Progression of the disease on imaging.
- Those who cannot tolerate toxicity.
- Subjects missed visits or had positive blood HCG;
- Other situations where the investigator felt it necessary to withdraw from the study.

# TERMINATION CRITERIA

- Unintended or unacceptable risks to the subject are identified;
- The study drug/trial treatment is ineffective, or it is pointless to continue the trial;
- For reasons such as difficulties in subject enrollment or frequent protocol deviations, the declarant decided to terminate the study.

# TRETAMENT REGIMEN AND DOSAGE

1. **Induction treatment stage: chemotherapy induction 2 cycles, q3w**

- Etoposide: 100 mg/m2 IV Day 1-3;
- Carboplatin: AUC 5 mg/mL/min IV Day 1

1. **Combination treatment stage: chemotherapy + immunotherapy + antivascular therapy 2-4 cycles, q3w**

- Etoposide: 100 mg/m2 IV Day 1-3
- Carboplatin: AUC 5 mg/mL/min IV Day 1
- Camrelizumab: 200 mg IV Day 1
- Apatinib: 250 mg PO QD

1. **Maintenance treatment stage: immunotherapy + antivascular therapy, q3w**

- Camrelizumab: 200mg IV Day 1
- Apatinib: 250mg PO QD

The treatment was administered in 3-week cycles until PD, intolerable toxicity, death, patient withdrawal of information, or discontinuation in the evaluation of the investigator.

# DOSE AJUSTMENT AND DISCONTINUATION OF ADMINISTRATION

## General Rules for Dose Adjustment

- Reasons for dose adjustments or delays in administration, measures taken, and results should be documented in the subject's medical record and electronic case report form (CRF).
- If concomitant symptoms were present at baseline, it was the investigator to decide whether the dose would be adjusted according to the level of change in the adverse effect. For example, a subject with a Grade 1 "weakness" status at baseline and a Grade 2 "weakness" status during the study treatment period may be considered a "weakness" condition. The dose should be adjusted for grade 1 toxicity.
- In the event that several adverse reactions of different grades or severity occur simultaneously, dose adjustments will be based on the highest grade observed.
- If a dose adjustment is required solely due to an abnormal laboratory hematology test, the dose will be adjusted based on the hematology test prior to the start of the treatment cycle.
- The current dose will be continued without dose adjustment or suspension of treatment when, in the evaluation of the investigator, the adverse reaction is unlikely to progress further to a serious or life-threatening event.
- If the toxicity is caused by another medication, no dosage adjustment of the other medication is necessary.
- Permanent discontinuation of one therapeutic agent prior to the onset of PD will not affect the continued use of other therapeutic agents.
- If one of the therapeutic medications is suspended due to an adverse reaction, but not all of the medications, the treatment is still counted as one treatment cycle.

## Camrelizumab Dose Adjustment

Increasing or decreasing the dose of camrelizumab is not permitted, only suspension or discontinuation. The maximum permissible suspension interval for camrelizumab is 6 weeks (counted from the time of the last actual dose), otherwise treatment with camrelizumab is discontinued. If the investigator determines that the subject would benefit from continued dosing with suspension of camrelizumab for more than 6 weeks, it requires discussion with the drug sponsor.

Refer to Table 1 for camrelizumab dose adjustment provisions and Annex VI for camrelizumab dose adjustment and toxicity management guidelines. Refer to Table 2 for management of infusion reactions associated with camrelizumab.

In addition, subjects were evaluated PD per RECIST v1.1 criteria should be permanently discontinued treatment. Subjects were allowed to discontinue medication due to other medical, or surgical, or accidental events unrelated to the study treatment. Subjects should resume the study treatment within 2 weeks after the interruption of medication, unless the investigator has other resolutions. The reasons for discontinuing treatment must be recorded in the CRF table.

**Table 1. Dose Adjustment Provisions for Camrelizumab**

| **Adverse reaction** | **Severity** | **Dosage adjustment** |
| --- | --- | --- |
| Pneumonia | Grade 2 | Suspension of drug administration ^a^ |
|  | Grade 3 or 4 | Permanent discontinuation |
| Diarrhea/ Colitis | Grade 2 or 3 | Suspension of drug administration ^a^ |
|  | Grade 4 | Permanent discontinuation |
| Dermatitis | Grade 3 | Suspension of drug administration ^a^ |
|  | Grade 4 | Permanent discontinuation |
| AST, ALT, or Bilirubin  Increased | Grade 2 | Suspension of drug administration ^a^ |
|  | Grade 3 or 4 | Permanent discontinuation |
| Hypophysitis | Grade 2 | Suspension of drug administration ^b^ |
|  | Grade 3 or 4 | Permanent discontinuation |
| Adrenocortical Insufficiency | Grade 2 | Suspension of drug administration ^b^ |
|  | Grade 3 or 4 | Permanent withdrawal |
| Hyperthyroidism | Grade 3 or 4 | Permanent discontinuation |
| Type I Diabetes | Grade 3 | Suspension of drug administration ^b^ |
|  | Grade 4 | Permanent discontinuation |
| Renal Insufficiency | Grade 2 or 3 | Suspension of drug administration ^a^ |
|  | Grade 4 | Permanent discontinuation |
| Neurotoxicity | Grade 2 | Suspension of drug administration ^a^ |
|  | Grade 3 or 4 | Permanent discontinuation |
| Infusion Reaction | Grade 3-4 | Permanent discontinuation |
| Other AE | Other Grade 3 AE first occurrence | Suspension of drug administration ^a^ |
|  | Grade 3 AE occurred again | Permanent discontinuation |
|  | Fail to drop to grade 0-2/baseline level within 7 days or 14 days.  Grade 3 AE within 0-1/baseline levels | Permanent discontinuation |
|  | Grade 4 AE | Permanent discontinuation ^c^ |

a: Resume dosing after symptoms recover to grade 0-1 or baseline level.

b: Hypophysitis, adrenocortical insufficiency, and type I diabetes mellitus may resume dosing when they are adequately controlled and require only physiologic hormone replacement therapy.

c: For Level 4 abnormal laboratory results, the decision to discontinue the medication should be based on concomitant clinical signs/symptoms and in accordance with the investigator's clinical evaluation.

**Table 2. Treatment Recommendations for Camrelizumab Infusion Reactions**

| **CTCAE Classification** | **Dosage Adjustment** | **Toxicity Treatment** |
| --- | --- | --- |
| Any Grade |  | Manage according to clinical practice.  Monitor subjects for infusion-related reactions (e.g., fever or chills, flushing and/or itching, changes in heart rate and blood pressure, dyspnea, chest discomfort, rash, etc.) and allergic reactions (e.g., generalized urticaria, rash, angioedema, asthma, hypotension, tachycardia, etc.) |
| Grade 1 | The infusion rate can be adjusted downward by 50% or temporarily neutralized. Discontinue infusion until infusion reaction subsides. | For Grade 1 or 2:  Acetaminophen and/or antihistamines given at the investigator's discretion according to clinical practice.  Consider prophylactic medication before subsequent regimens according to local clinical practice. |
| Grade 2 | The infusion rate can be reduced by 50% or the infusion can be temporarily interrupted until the infusion response subsides.  Infusion rate adjustable to 50% of initial rate after that. |  |
| Grade 3/4 | Permanent discontinuation. | For Grade 3 or 4:  Manage severe infusion-related reactions according to local clinical practice (e.g., administer epinephrine, iphenhydramine, ranitidine and glucocorticoids) |

## Apatinib Dose Adjustment

The initial dosage of apatinib is 250mg once daily (250mg qd). If intolerable adverse effects occur, the dosage can be adjusted to 250mg every other day (250mg qod). If intolerance persists, discontinue the medication until symptoms reduce to Grade 1 or lower, then resume at 250mg qd. Permanent discontinuation is recommended if Grade 3 or higher adverse effects recur.

During treatment, suspension of administration is required for reasons of non-recovered drug toxicity, and the cumulative duration of dosing suspension should not exceed 2 weeks per dosing cycle to ensure that the subject receives the drug strength of the treatment. If the investigator determines that an adverse reaction is caused by apatinib, apatinib dosing may be suspended without affecting the use of other medications. Refer to Table 3 for apatinib dose adjustment.

**Table 3. Dose Adjustment Provisions for Apatinib**

| **NCI Toxicity Classification** | **Dose Adjustment for Hematologic Toxicity** |
| --- | --- |
| Grade 1, 2 Hematologic Toxicity | Maintain original dose level (appropriate symptomatic treatment) |
| Grade 3 Hematologic Toxicity | Firstly, suspend the drug and start symptomatic treatment, wait for the toxicity to recover to ≤ grade 2, then continue the treatment at the original dose |
| Grade 4 Hematologic Toxicity | Firstly, suspend the drug and start symptomatic treatment, wait for the toxicity to recover to ≤ grade 2, then continue the treatment at the original dose |
| **Dose Adjustment for Non-Hematologic Toxicity** | |
| Grade 1 Non-Hematological Toxicity | Maintain original dose level (appropriate symptomatic treatment) |
| Grade 2 Non-Hematological Toxicity | Symptomatic treatment, if the toxicity returns to ≤ grade 1 after two weeks, continue the drug; If the toxicity does not aggravated, suspend the drug and wait until the toxicity recovers to ≤ grade 1, continue to the original dose level. |
| Grade 3 Non-Hematological Toxicity | Suspend the drug and start symptomatic treatment, and continue treatment at the original dose when the toxicity recovers to ≤ grade 1; If grade 3 occurs again then discontinue related medication treatment. |
| Grade 4 Non-Hematological Toxicity | Suspend the drug and start symptomatic treatment, and continue treatment at the original dose when the toxicity recovers to ≤ grade 1. If it reccurs Grade 4 toxicity, terminate related drug therapy. (If life-threatening adverse events occur such as Grade4 renal damage, neurotoxicity, cardiotoxicity, hepatotoxicity, etc., terminate related drug therapy immediately) |
| Adverse reactions requiring special attention: In the event of hypertensive crisis, cerebral hemorrhage, ≥ Grade 2 pulmonary hemorrhage, ≥ Grade 3 other bleeding, arterial thrombosis, Grade 4 venous thrombosis, leukoencephalopathy syndrome, or gastrointestinal perforation, discontinue the administration of drugs associated with adverse effects and initiate prompt symptomatic treatment. | |

## Chemotherapy Dose Adjustment

The doses of carboplatin and etoposide may be adjusted based on the prescribing information and local treatment standards due to toxicities. The guideline for dose modifications is provided below. Once the dose is reduced, it cannot be increased back to 100% of the original dose. The treatment with carboplatin or etoposide should be discontinued if any Grade 3 or 4 hematologic or non-hematologic toxicity occurs after 2 dose reductions or a treatment delay of more than 9 weeks due to toxicity.

**Hematologic toxicity**

The ANC should be ≥ 1500/μL and the platelet count should be ≥ 100,000/μL before starting each cycle. In order to provide sufficient time for recovery, the treatment can be delayed for up to 9 weeks. After 2 dose reductions or treatment delays exceeding 9 weeks due to toxicity, treatment with etoposide and/or carboplatin was discontinued unless the investigator believed that patients could benefit from continued treatment. Growth factor therapy may be provided as per the American Society of Clinical Oncology (ASCO) and NCCN guidelines (Smith et al. 2006; NCCN 2015). At the beginning of subsequent cycles after recovery, the dose should be modified based on the PLT and ANC nadirs of the last cycle (refer to Table 4). Subjects who require dose modification due to both ANC and PLT should receive a lower dose.

**Table 4. Chemotherapy Dose Modification due to Hematologic Toxicity**

| **Toxicity**^a^ | **Carboplatin Dose** |
| --- | --- |
| ANC < 500/μL and PLT ≥ 50,000/μL | 75% of previous dose^b^ |
| PLT < 25,000/μL, ANC not considered | 75% of previous dose^b^ |
| PLT < 50,000/μL with Grade ≥ 2 hemorrhage, ANC not considered | 50% of previous dose^b^ |
| ANC < 1000/μL with fever ≥ 38.5 °C | 75% of previous dose^b^ |

a: Nadir of the last treatment cycle. b: The calculated dose before dose administration of each cycle.

**Non-hematologic toxicity**

Treatment should be delayed (up to 9 weeks) if any non-hematologic toxicity occurs until the value returns to baseline or below baseline (or Grade ≤ 1 if the subject has no toxicities at baseline). At the beginning of the subsequent cycles, the dose should be reduced based on the non-hematologic toxicity observed from the last cycle. Table 5 provides the dose modification recommendations for non-hematologic toxicity.

**Table 5. Dose Modification or Treatment Discontinuation due to Non-Hematologic Toxicity**

| **Toxicity** | **Modified Carboplatin Dose (by % of Previous Dose)**^a^ | |
| --- | --- | --- |
| Diarrhea | Grade 3 or 4^b^ | 75% |
| Nausea/Vomiting^C^ | Grade 3 or 4 | 75% |
| Neurotoxicity | Grade 2 | 75% |
| Grade 3 or 4 | 50% or permanent discontinuation |  |
| AST, ALT, or Bilirubin  Increased | Grade 3 | 75% |
| Grade 4 | Discontinuation |  |
| Others | Grade 3 or 4 | 75% |

a The carboplatin dose can be modified by a specific percentage of the original AUC if deemed appropriate by the investigator. Previous dose: The calculated dose before dose administration of each cycle.
b Antidiarrheal agents are used for Grade 3-4 diarrhea or for any grade of diarrhea that requires hospitalization.
c Even when antiemetic therapy has been given.

Diarrhea should be treated with appropriate antidiarrheal agents. Nausea and/or vomiting should be treated with appropriate anti-emetics. If Grade 3 or 4 nausea/vomiting occurs, anti-emetics should be used and the dose should be reduced by 25% in the next cycle. The original dose should be resumed as soon as the subject is able to tolerate. If Grade 3 or 4 neurotoxicity occurs,chemotherapy should continue at 50% of the previous dose after improvement, or immediately be discontinued (based on the investigator's judgment).

**Table 6. Dose Modifications of Etoposide for Subjects with Renal Impairment.**

| **Creatinine Clearance (mL/min)** | **Etoposide Dose** |
| --- | --- |
| > 50 | 100% |
| 15-50 | 75% of previous dose^a^ |

a: The calculated dose before dose administration of each cycle.

# CONCOMITANT THERAPY

## Hematologic Support

Colony-stimulating factor (G-CSF or GM-CSF) or erythropoietin may be used prophylactically during treatment to prevent possible adverse events such as neutropenia.

## Treatment of Symptoms of Non-Hematologic Toxicity

Supportive therapies such as antiemetics, antibiotics, pain medications, blood pressure medications, and blood products may be used. Rinses can be used to treat or prevent stomatitis.

Unconventional therapy treatments (e.g., herbs or acupuncture) and vitamins/minerals may be used if the investigator believes there is no effect on the study's endpoints. Patients may receive bisphosphonates for bone metastases during treatment. If painful bone metastatic lesions are not effectively controlled by systemic therapy or local analgesia, small-area (radiotherapy area must be <5% bone marrow area) irradiation with palliative radiotherapy is permitted. Clinical co-morbidities and the presence of various types of AE should be actively treated and managed. All drugs used in combination should be recorded in the case report form (CRF) in strict accordance with GCP.

## Other Anti-tumor/Cancer or Investigational Drugs

Subjects are prohibited from receiving the following treatments during the treatment period of this study:

- Systemic chemotherapy and biologic therapy (including antitumor agents with immunomodulatory effects, including but not limited to, e.g., interferon, interleukin-2, thymosin, immune cell therapy, etc.).
- Immunotherapy not specified in this protocol.
- Drugs other than those specified in this study, including proprietary Chinese medicines with clear antitumor indications.
- Radiation therapy (Note: Sponsor may approve and allow radiation therapy to symptomatic isolated lesions or to the brain as long as it is not a lung or target lesion.).
- Live vaccines, including, but not limited to, the following: measles, mumps, rubella, varicella, yellow fever, rabies, BCG, and typhoid (oral) vaccines, within 28 days prior to the first dose of study medication of the study treatment and during participation in the study. Inactivated viral vaccines for seasonal influenza, administered by injection, are permitted; however, live attenuated influenza vaccines administered intranasally are not permitted.
- Corticosteroids, Inhaled steroids allowed for use in the treatment of asthma or chronic obstructive pulmonary disease (COPD) as part of stationary therapy. Corticosteroids for the management of potential irAE are allowed. Physiologic doses of corticosteroids are allowed. Allow prophylactic corticosteroids to avoid allergic reactions (e.g., intravenous contrast media).

Subjects requiring any of the above treatments, as assessed by the investigator, should be excluded from the study. Subjects may receive other medications as deemed medically necessary by the investigator. It is important for the investigator to review each medication (prescription and non-prescription) received by the subject prior to study entry and at each study visit.

At each visit, subjects must be asked about any new medications they have received since the previous visit;

In order to reduce the risk of drug interactions, all measures must be taken to limit the number of concurrent administrations that are truly necessary;

Drugs that are hepatotoxic (i.e., drugs with warnings of hepatotoxicity in the product insert) should be avoided during administration. Investigators are encouraged to review the potential hepatotoxicity of each drug by searching the [www.livertox.nih.gov](http://www.livertox.nih.gov/) website.

# STUDY PROCEDURES

Each patient has to go through three phases to complete this clinical study after signing the informed consent form: screening period, treatment period, and follow-up period. Before starting the study, patients must read and sign the Ethics Committee (EC)-approved informed consent form. All study steps were required to be performed within the time window specified in the trial flow chart. All observations and examination times are independent of the length of discontinuation of the drug, and the relevant examinations will be performed at each session as specified.

## Screening Period

**The investigator will enroll subjects as follows.**

Obtain an ICF with the subject's signature prior to any study-related processes. Subjects were formally determined to be eligible for enrollment after review of the inclusion/exclusion criteria by the principal investigator or a designated person authorized by the principal investigator with appropriate training.

Subjects who do not meet the relevant criteria for this study (screening failure) may receive rescreen. Each subject may be rescreened once again. At the time of rescreening, the subject must re-sign the ICF and will be reassigned a subject identification number.

If a subject is discharged from the group or is ineligible for enrollment due to early onset of PD, withdrawal of ICF, or intolerance without 28 days of observation, the principal investigator of the group leader unit will decide whether or not to replenish the subject, as appropriate.

**Procedures for incorrectly enrolled subjects**

Enrollment criteria must be strictly adhered to. If a subject is found to be enrolled who does not meet the enrollment criteria, the Principal Investigator is required to discuss and determine whether or not to continue the subject's participation in this study with or without the study drug. If the investigator determines that it is medically appropriate for the subject to remain in the study and the drug provider agrees with the investigator's decision, the subject may continue to participate in the study and receive the study drug. If the Investigator determines that it is medically appropriate for the subject to remain in the study, but the drug supplier disagrees with the Investigator's determination, the subject may not continue to participate in the study (with or without treatment with the study drug). Refer to Table 7 for the screening visit process.

**Table 7. Screening Visits Process**

| **Screening period** | **-28-1 days** | **-7-1 days** | **Note** |
| --- | --- | --- | --- |
| Baseline | | | |
| Written Informed Consent Form | X |  | For those who did not meet the relevant criteria for this study subjects may be rescreened. |
| Inclusion/Exclusion Criteria | X |  | Refer to section 5.1 and 5.2 |
| Demographics/Medical History/Previous Medications | X |  | Prior medications include treatments for the initial diagnosis, including chemotherapy, radiation therapy, and surgical treatments, the time of the latter antitumor treatment must be recorded. |
| Vital Signs | X |  | Vital signs include: temperature, pulse, respiratory rate and blood pressure. |
| Weight, Height | X |  | Refer to section 13.1.2 |
| Physical Examination | X |  | Refer to section 13.1.2 |
| ECOG PS Sore | X |  | Refer to section 13.1.2 |
| Laboratory tests | | | |
| 12-lead ECG | X |  | Refer to section 13.1.2 |
| Hematology/Blood Biochemistry Urinalysis/Urine  Routine/Stool Tests |  | X | Refer to section 13.1.1 |
| Coagulation Function |  | X | Refer to section 13.1.1 |
| Pregnancy Test |  | X | Refer to section 13.1.2 |
| Thyroid Function Test | X |  | Refer to section 13.1.2 |
| Myocardial Enzyme Test |  | X | Refer to section 13.1.2 |
| Pituitary-adrenal axis Test | X |  | For hospitals where conditions permit |
| HIV, HBV and HCV Test | X |  | Refer to section 13.1.2 |
| Echocardiography |  | X | Refer to section 13.1.2 |
| Pulmonary Function Tests and Arterial Blood Gas (ABG) Analysis | X |  | Refer to section 13.1.2 |
| AE Assessment | X |  | The safety assessments for AEs and laboratory tests will be performed according to NCI CTCAE v5.0. All AEs that occur after signing the informed consent form to 30 days after the last dose must be monitored and ocumented. If a subject starts a new anti-tumor treatment during the AE collection period, then only treatment-related AEs should be collected after the start of new anti-tumor treatment. |
| Concomitant Medications | X |  | All concomitant medications/treatments from 28 days prior to study treatment until the end of the safety follow-up period should be collected. If a subject starts a new systemic anti-tumor treatment during the study, then only concomitant medications/treatments used for study treatment-related AEs should be documented. |
| Evaluation of Efficacy | | | |
| Imaging Evaluation | X |  | Refer to section12.3 and 12.4 |

## Treatment period

Subjects who met the inclusion criteria and did not meet any of the exclusion criteria and signed the informed consent form were enrolled in the treatment period after passing the baseline examination. The investigator will administer the study drug according to the protocol and will monitor the subject for any adverse reactions and efficacy, which will be promptly recorded in the original medical record. Refer to Table 8 for the flow of visits during the treatment period.

**Table 8. Treatment Visits Process**

| **Treatment Period** | **Cycle 1**  **D1** | **Cycle 2**  **D1±3** | **N^th^ cycle**  **D1±3** | **Note** |
| --- | --- | --- | --- | --- |
| Laboratory Tests and AE Assessment | X | X | X | All AEs that occur after signing the informed consent form to 30 days after the last dose must be monitored and documented. If a subject starts a new anti-tumor treatment during the AE collection period, then only treatment-related AEs should be collected after the start of new anti-tumor treatment. |
| Concomitant Medications | X | X | X | All concomitant medications/treatments from 28 days prior to study treatment until the end of the safety follow-up period should be collected. If a subject starts a new systemic anti-tumor treatment during the study, then only concomitant medications/treatments used for study treatment-related AEs should be documented. |
| Evaluation of Efficacy | | | | |
| Imaging Evaluation |  | X | X | Refer to section12.3 and 12.4 |
| Infusion of Study Drugs | | | | |
| Camrelizumab | 200mg IV, Q3W | | | Refer to section 8. |
| Apatinib | 250mg PO QD | | | Refer to section 8. |
| Carboplatin | AUC 5 mg/mL/min IV Q3W | | | Refer to section 8. |
| Etopside | 100 mg/m2 IV Day 1-3 Q3W | | | Refer to section 8. |

## End of Treatment

End of treatment refers to withdrawal from the study. An end-of-treatment visit is required ± 3 days from the time of the decision to terminate treatment and/or withdraw from the study. Refer to Table 9 for the flow chart for end-of treatment visits.

**Table 9. End-of-Treatment Visits Process**

| **End of Treatment** | **±3 days** | **Note**  **21 days (±7 days)** |
| --- | --- | --- |
| Vital Signs | X | Vital signs include: temperature, pulse rate, respiratory rate, and blood pressure.  Refer to section 13.1.2 |
| Weight | X | Refer to section 13.1.2 |
| Physical Examination | X | Refer to Program 13.1.2 |
| ECOG PS Sore | X | Refer to section 13.1.2 |
| Laboratory Tests | | |
| 12-lead ECG | X | Refer to section 13.1.2 |
| Hematology/Blood Biochemistry/Urine /Stool Test | X | Refer to section 13.1.1 |
| Coagulation | X | Refer to section 13.1.1 |
| Pregnancy Test | X | Refer to section 13.1.2 |
| Thyroid Function | X | Refer to section 13.1.2 |
| Myocardial Enzyme Test | X | Refer to section 13.1.2 |
| Pituitary-adrenal axis Test | X | For hospitals where conditions permit. |
| Echocardiography | X | Refer to section 13.1.2 |
| Pulmonary Function Tests and Arterial Blood Gases |  | When clinically indicated in the judgment of the investigator. |
| AE Assessment | X | The safety assessments for AEs and laboratory tests will be performed according to NCI CTCAE v5.0. All AEs that occur after signing the informed consent form to 30 days after the last dose must be monitored and documented. If a subject starts a new anti-tumor treatment during the AE collection period, then only treatment-related AEs should be collected after the start of new anti-tumor treatment. |
| Concomitant Medications | X | All concomitant medications/treatments from 28 days prior to study treatment until the end of the safety follow-up period should be collected. If a subject starts a new systemic anti-tumor treatment during the study, then only concomitant medications/treatments used for study treatment-related AEs should be documented. |
| Evaluation of efficacy | | |
| Imaging Evaluation | X | Refer to section12.3 and 12.4 |
| Biomarker Exploration | | |
| Archived or Fresh Tumor Tissue Specimens | X | At least 10 white sheets 4-6 μm thick (optional) |
| Peripheral Blood Test | X | 16 mL of peripheral blood was taken for tumor marker testing. |

## Safety Follow-Up

Safety follow-up will be conducted 90±7 days after the last dose. Refer to Table 10 for safety follow-up process.

**Table 10. Safety Follow-up Process**

| **Safety Follow-Up** | **42±7 days** | **Note** |
| --- | --- | --- |
| Vital Signs | X | Vital signs include: temperature, pulse rate, respiratory rate and blood pressure.  Refer to 13.1.2 |
| Weight | X | Refer to 13.1.2 |
| Physical Examination | X | Refer to section 13.1.2 |
| ECOG PS Score | X | Refer to section 13.1.2 |
| Laboratory Tests | | |
| 12-lead ECG | X | Refer to section 13.1.2 |
| Hematology/Blood Biochemistry/Urine /Stool Test | X | Refer to section 13.1.1 |
| Coagulation Function | X | Refer to section 13.1.1 |
| Thyroid Function Test | X | Refer to section 13.1.2 |
| AE Assessment | X | All AEs that occur after signing the informed consent form to 30 days after the last dose must be monitored and documented. If a subject starts a new anti-tumor treatment during the AE collection period, then only treatment-related AEs should be collected after the start of new anti-tumor treatment. |
| Concomitant Medications | X | All concomitant medications/treatments from 28 days prior to study treatment until the end of the safety follow-up period should be collected. If a subject starts a new systemic anti-tumor treatment during the study, then only concomitant medications/treatments used for study treatment-related AEs should be documented. |
| End-of-Treatment Visits | | |
| State of Survival | X | Once every 3 months after finished treatment. |
| Follow-up Anti-Tumor Therapy | X |  |
| Evaluation of Efficacy | | |
| Imaging Evaluation | X | For discontinuation of therapy for reasons other than imaging PD, imaging evaluations will be performed according to the imaging evaluation time cutoffs, to the extent possible, until either of the following events occurs: initiation of a new antitumor therapy, PD, subject withdrawal of ICF, and death. |

# CLINICAL EVALUATION

## Primary Efficacy Indicators

- Safety : Evaluated according to CTCAE 5.0 criteria.

## Secondary Efficacy Indicators

- Progression-free survival (PFS)：PFS was determined as the interval from initiation of the study dose to disease progression or death, whichever occurred first.
- Overall survival (OS): OS was determined as the interval from initiation of the study dose to death from any cause.
- Overall survival (ORR): ORR was determined as the percentage of patients who had complete response (CR) or partial response (PR) as best overall response per RECIST v1.1.
- Disease control rate (DCR): DCR was defined as the percentage of patients with CR, PR or SD. DoR was determined as the interval from the initial documented objective response to disease progression or death from any cause.

## Imaging Scan Site

The method used for tumor imaging evaluation at baseline must be consistent with the method used for each subsequent follow-up evaluation, and a computed tomography (CT) or magnetic resonance imaging (MRI) scan is recommended. Other sites of involvement are examined as prompted by each subject's symptoms and signs. Thoracic, abdominopelvic (scans from the apex of the lungs to the suprapubic symphysis), brain, and bone scans, as well as all known or suspected sites of disease, must be performed at baseline. Each subsequent clinical tumor imaging evaluation should include thoracic abdominal and pelvic, and may include brain and/or bone scans when clinically indicated. Investigators may increase the frequency of imaging surveillance as appropriate.

## Evaluation Time Point

Baseline tumors will be performed within 28 days prior to the first dose, and imaging obtained prior to signing informed consent may be used for screening phase tumor assessment as long as it meets protocol requirements. Imaging evaluations of C1D21 and C1D21 were performed every 6 weeks (±7 days) thereafter until imaging PD was recorded.

If the investigator is unable to determine whether the disease has progressed, especially if there is uncertainty about the certainty of non-target lesions and new lesions, the subject may continue treatment and the subject's disease status may be reassessed when clinical indication occurs, or at the next scheduled evaluation time point. The date of progression should be the date of initial detection if PD is confirmed on scan review.

Suspension of one or more study drugs does not affect the frequency of RECIST v1.1 assessments.

# SECURITY ASSESSMENT

## Security Indicators

### Indicators for routine laboratory safety assessment

**Table 11. Routine Laboratory Safety Assessment**

| Hematology Test | RBC, HGB, HCT, WBC, PLT, LYM, ANC, MONO, EOS and BASO |
| --- | --- |
| Coagulation Function | TT, PT, APTT and INR |
| Blood Biochemistry | TBIL^a^, ALT, AST, γ-GT , ALP, ALB, TP, LDH, BUN, Cr, Na, K,  Cl, Mg, Ca, P, lipase, amylase, and FBG |
| Urine Routine | PH, UALB, UPRO^b^, URBC^c^ and UGLU |
| Stool Test | occult blood (in medicine, fecal blood from internal bleeding) |

1. If TBIL ≥ 2 × ULN (and there is no evidence of Gilbert's syndrome), then direct and indirect bilirubin were measured separately.
2. Leukocytes should be examined microscopically (if appropriate) and erythrocytes should be examined with a high magnification field of view.

### Physical examination

A complete physical examination includes: general condition, respiratory, cardiovascular, abdominal, skin, head and neck (including ears, eyes, nose, and throat), lymph nodes, thyroid, musculoskeletal (including spine and extremities), genital/anal, and neurologic evaluation.

1. **12-lead electrocardiogram**

Resting 12-lead ECGs will be analyzed at a local laboratory according to the visit schedule. A 12-lead ECG should be performed in each case after the subject has rested in the prone position for at least 5 minutes, and further ECGs will be performed when clinically indicated cardiac-related event. The investigator completes the ECG assessment on the day of the examination and records the results on the ECG. The same assessment should be used throughout the study.

ECGs will be recorded at 25 mm/sec. The investigator should evaluate all ECGs according to the category of clinically significant abnormality/non-clinically significant abnormality; if there is a clinically significant abnormality results, the investigator should document the result as an AE in the CRF.

1. **Vital signs**

Vital signs will be checked as described in the study visit schedule. Vital signs include temperature, pulse rate, respiratory rate and blood pressure.

Additional vital sign assessment monitoring may be performed at the discretion of the investigator in accordance with standard clinical practice or based on clinical need.

In the presence of AE/SAE, additional recorded vital sign values may be taken on the CRF (if applicable).Record the date and time of collection and measurement in the appropriate section.

- 1. **Pulse and blood pressure**

Pulse and blood pressure were taken during the Screening Period and prior to the planned administration of the daily infusion of Camrelizumab.

Blood pressure monitoring: For each blood pressure measurement, no smoking or coffee for 30 minutes prior to the measurement and rest quietly for at least 10 minutes.

Minutes were taken in a sitting position with the elbow placed at the same level as the heart, and each blood pressure measurement was taken on the same side.

Blood pressure was measured by the investigator during the screening period and prior to each scheduled administration of camrelizumab; during the study, blood pressure monitoring was done by the subjects themselves and recorded on subject diary cards, and blood pressure was measured weekly for the first 2 cycles.

- 1. **Body temperature and breathing**

Temperature and respiration were taken during the screening period and prior to the planned administration of the daily infusion of camrelizumab.

- 1. **Height and weight**

Height measurements were taken only during the Screening Period, and weight measurements were required during the Screening Period, prior to the administration of camrelizumab at each visit, at the end of treatment, and at the safety follow-up visit.

- 1. **Pregnancy test**

Women of childbearing potential undergo a serum human chorionic gonadotropin (hCG) sample pregnancy test within 7 days prior to the first dose of study drug. If the result is positive, the subject is ineligible for enrollment and must discontinue participation in the study. Pregnancy suspected to have occurred during the study should be reviewed.

- 1. **Other tests**
- Hepatitis B tests: HBsAg, HBsAb, HBcAb, HBeAg, HBeAb.
- HIV antibody, HCV antibody tests.
- Thyroid function tests: T3, T4, TSH, FT3, and FT4.
- Myocardial enzyme profile tests: LDH, AST, CK, CK-MB and ALT.
- Echocardiography.
- Lung function tests: VT, BE, MV, ERV, IC, VCmax, FVC, FEV1, FEV1/FVC, PEF, MEF75, MEF50, MEF25, MVV, TLC, RV/TLC, BR, DLCO SB, Raw, eff and sGaw eff.
- Arterial blood gas analysis: including Ph, PaCO2, AB, SB, BB, BE, TCO2, CO2-CP, PaO2, and SaO2 metrics were assessed.

## Adverse Event

Adverse Event (AE) Definition: an AE is any unfavorable medical event that occurs after a subject or clinical trial participant receives a drug or treatment regimen that is not necessarily causally related to the treatment.

AE can be any sign (including abnormal laboratory test results), symptom, or disease that is unpleasant or unrelated to the purpose for which the product was used, whether or not it is considered to be related to the medical product, that is temporally related to the use of the medical product.

Events that occur during the pre- and post-treatment phases are also considered AEs under the regulations. Therefore, safety monitoring of AE or reporting of SAEs should begin once the subject participants in(signs the informed consent form) and continue through the end-of-trial visit.

## AE Classification

AEs are categorized as grades 0-5 according to the NCI-CTCAE 5.0. Classification Criteria. If the NCI AEs not listed in the toxicity grading criteria may be judged according to the following criteria:

- Grade 1: Mild, no clinical symptoms or cyanotic clinical symptoms; only clinical or laboratory test abnormalities; no treatment required; does not affect normal daily activities;
- Grade 2: Moderate, requiring minimal, localized or non-invasive treatment; interferes with normal daily activities (cooking, shopping, talking on the phone, counting money, etc.)
- Class 3: Serious or medically serious symptoms that are not life-threatening for the time being; result in hospitalization or prolonged hospitalization; result in disability; inability to work or perform normal daily activities (bathing, dressing, undressing, eating, going to the bathroom, taking medication, etc.), non-bedridden;
- Grade 4: life-threatening, requiring urgent treatment;
- Grade 5: death due to adverse event.

## AE Records

The name, severity, time of presentation, duration, management measures, and regression of various AEs occurring during the trial were recorded in detail and faithfully filled in the case report form (CRF). Abnormal laboratory test data should be recorded in the CRF. The test was repeated at least once a week until normalization or the end of the study. 30 days after the last dose of

Adverse events that occur within days are reported and recorded.

## AE Causality Assessment with Investigational Medicinal Product

Possible associations between AEs and the test drug were assessed according to a five-level classification of "definitely related, highly likely related, possibly related, possibly unrelated, and unrelated" (see Table 12.)

The levels of “definitely related, highly likely related, possibly related” were considered drug-related. The following table shows the criteria for determining the relationship between AE and drug:

**Table 12. Criteria for Determining the Relationship between AE and Medications**

|  | Definitely Related | Highly likely Related. | Possibly Related | Possibly Unrelevant | Unrelated |
| --- | --- | --- | --- | --- | --- |
| Reasonable chronology | √ | √ | √ | √ | × |
| Types of reactions to known drugs | √ | √ | √ | × | × |
| Removing the cause can improve | √ | √ | √ or × | √ or× | × |
| Re-administration can be repeated | √ | ? | ? | ? | × |
| There may be another explanation for the reaction | × | × | × | √ | √ |

## Serious Adverse Events SAE

1. Serious adverse event (SAE) is a medical event that occurs during the course of a clinical trial that requires hospitalization or prolonged hospitalization, is disabling, affects the ability to work, is life-threatening or death-threatening, or results in a congenital malformation. Includes the following unintended medical events: events that result in death; life-threatening events (defined as a subject being in danger of death at the time of the event); events that require hospitalization or extended hospitalization; events that can result in permanent or severe disability/insufficiency; and carcinogenicity or teratogenicity.
2. Drug exposure during pregnancy/lactation. In principle, pregnancy and lactation are inclusion exclusion criteria. If pregnancy occurs during the study, then the patient should be withdrawn from the study immediately, the investigator should be informed immediately, and the patient should be followed throughout the pregnancy and postpartum. The consequences should be documented even if both mother and child are completely normal without any adverse events. Even if the pregnancy is not an SAE, it should be reported using the SAE reporting form.
3. Disease progression (including signs and symptoms of progression) should not be reported as a Serious Adverse Event, but death due to disease progression should be reported as a Serious Adverse Event if it occurs within the trial or safety reporting period. Hospitalization for signs and symptoms of disease progression should not be reported as a Serious Adverse Event. If the final outcome of the cancer is death during the trial or safety reporting period, then the event leading to death must be reported as a serious adverse event.
4. Administering other antitumor therapy: If the subject is initiating other antitumor therapy, the reporting period for adverse events that are not deaths is up to the start of the new antitumor therapy. If the death occurs within the Serious Adverse Event Reporting Period after the end of study treatment, it must be reported regardless of whether the patient receives other treatment.
5. Hospitalization: Adverse events resulting in hospitalization or prolonged hospitalization in a clinical study should be considered serious adverse events. Any initial admission to a healthcare facility (even if shorter than 24 hours) meets this criterion. Hospitalization does not include the following: rehabilitation facilities, nursing homes, routine emergency room admissions, same-day surgery, hospitalizations or prolonged hospital stays not associated with a worsening of an adverse event that are not serious adverse events per se, (e.g., for the purpose of checking for abnormalities in laboratory tests that persisted to date prior to the trial);hospitalization for administrative reasons (e.g., routine annual physical examination); hospitalization mandated by the trial protocol during the clinical trial (e.g., performed as required by the trial protocol); elective hospitalization unrelated to the worsening of an adverse event (e.g., elective cosmetic surgery); scheduled treatments or surgeries that should have been documented throughout the trial protocol and/or in the individual subject's baseline data; hospitalization for the sole reason of blood product Admission for Use. Diagnostic or therapeutic invasive (e.g., surgery), non-invasive procedures should not be reported as adverse events. However, when the disease condition that led to the procedure meets the definition of an adverse event, it should be reported, e.g., acute appendicitis that developed during the adverse event reporting period should be reported as an adverse event, and the resulting appendectomy should be documented as the treatment for that adverse event.
6. Overdose: An overdose is defined as the addition of a trial medication by a subject within 24 hours (time adjusted to the specific protocol) at a dose higher than that prescribed by the investigator's physician. All trial drug overdoses should be reported as serious adverse events, whether or not they are related to an adverse event/serious adverse event.

## SAE's Reporting Procedures

Serious adverse events should be reported from the time the subject signs the informed consent form up to and including the 30th calendar day after the last dose of study drug. During the trial period, if a serious adverse event occurs, it must be reported to the clinical supervisor and the principal investigator within 24 hours, and at the same time fill in the "Serious Adverse Event (SAE) Report Form ", sign and date it.

All serious adverse events must be reported. Serious adverse events occurring more than 30 days after the last dose are generally not reported unless they are suspected to be related to the study drug.

Serious adverse events should be recorded in detail with symptoms, severity, time of occurrence, time of treatment, measures taken, and follow-up time and modality and regression. If the investigator believes that a serious adverse event is not related to the test drug but is potentially related to a study condition (e.g., termination of the original treatment, or comorbidities during the course of the trial), this relationship should be detailed in the narrative portion of the Serious Adverse Event page of the Medical Record Report Form. If the intensity of a particular ongoing serious adverse event or its relationship to the subject drug changes, a follow-up report of the serious adverse event should be sent immediately. All serious adverse events should be followed until recovery or stabilization.

# DATA COLLECTION AND DATA MANAGEMENT

## Data Auditing and Monitoring

Data entry and management is done by an independent data manager. For queries present in the case report form, the data manager will write a question answer form (DRQ) and send a query to the investigator through the clinical supervisor, who should answer and return as soon as possible, and the data manager will make data corrections, confirm and enter data based on the investigator's answers, and may send another DRQ if necessary.

## Database lockout

After data review and confirmation, the database are lockouted by the principal investigator, the statistical analyst. No further changes are made to the data files after locking.

## Statistical Analysis of Data Sets

Full Analysis Set (FAS): the efficacy of all cases enrolled and using the drug at least once was analyzed according to the principle of Intentional Analysis (ITT).

Efficacy Analysis Set (EAS): Patients who patients who received at least one dose of study drug and had at least one post-treatment tumor evaluation constituted the evaluable analysis set (EAS) population.. The efficacy of the drugs was statistically analyzed by both FAS and PPS.

Safety Analysis Set (SAS): all enrolled cases, all patients who have used the trial drug at least once and have a post-dose safety record. This data set is used for safety analysis.

## Statistical analysis plan

The results of this trial were mainly described statistically. Continuous data are presented with mean, standard deviation, median, maximum, and minimum values, while count data and ordinal data are presented with frequency (percentage), rate, and confidence interval. All statistical analyses will be programmed and calculated using SAS 9.3 statistical analysis software OR R version 3.4.1. All statistical tests will be two-sided, with a p-value less than or equal to 0.05 being considered statistically significant, and 95% confidence intervals will be used for the differences tested.

Basic patient characteristics are presented with mean, standard deviation, median, maximum and minimum values for quantitative data, and frequencies and percentages were listed for qualitative data.

Efficacy analysis: median PFS and its 95% CI were calculated using the kaplan-Meier method and their survival graphs were plotted for comparison. For secondary efficacy indicators, the kaplan-Meier method was used to estimate the median OS and its 95% CI, and survival graphs were plotted. Objective remission rate (ORR=CR+PR) and disease control rate (DCR=CR+PR+SD) were calculated and their 95% CIs. Quality of life scores were compared with baseline values using paired t-tests or signed rank-sum tests to compare the before-and-after differences within groups.

Safety evaluation: descriptive statistical analysis is the main focus, the table describes the adverse events and adverse reactions that occurred in this trial. (where an adverse reaction is defined as “an adverse event that is 'definitely related/highly likely related/possibly related' to the study drug”) Laboratory test results describing pre-test normal but post-treatment abnormalities and the relationship to the test drug when abnormal changes occurred.

# MEDICINES MANAGEMENT

The management, distribution and recovery of the drugs used in this clinical trial are under the responsibility of specialized personnel, and the test drugs should be sealed, protected from light, and stored at the proper temperature. The investigator must ensure that all test drugs are used only for the subjects participating in the clinical trial, and their dosage and usage should follow the trial protocol, and the remaining drugs are returned to Jiangsu Hengrui Pharmaceutical Co., Ltd. and the test drugs for clinical use shall not be transferred to any non-clinical trial participants.

When the drug is distributed, a drug receipt form shall be signed, double signed, in duplicate, one for each of the clinical research unit and Jiangsu Hengrui Pharmaceutical Co. At the end of the study, the remaining drugs and empty boxes will be collected. Both parties shall sign the drug recovery form. The issuance and recovery of each drug shall be recorded in a timely manner on a special record sheet. Supervisors are responsible for supervising the process of supplying, using, storing and disposing of the remaining drugs used in the clinical trial.

Upon written notification to the Co-organizer, the investigator may destroy used drug products and empty cartridges, provided that the health of the population is not jeopardized. The investigator should maintain records of the disposal of all trial medications. These records must show the certification and quantity of drugs destroyed in each batch, as well as the method of disposal (in accordance with the requirements of local law), and the person who disposed of the drugs.

# ETHICAL CONSIDERATIONS

The study was conducted in strict compliance with the ethical guidelines for human medical research of the Declaration of Helsinki and Chinese clinical trial regulations. Prior to the commencement of the study, the protocol and subject informed consent forms were reviewed and approved by the PI unit and the Ethics Committee prior to the commencement of the study.

During the study period, it was strictly ensured that all operations were carried out in accordance with the approved protocols During the implementation process, any modification of the study protocols had to be re-submitted to the Ethics Committee for consent, and the occurrence of severe adverse events was reported to the Hospital Ethics Committee.

Before each subject was enrolled in this study, a complete, comprehensive and truthful description of the purpose, steps and possible risks, benefits and related rights and interests of the study was given to the affected person or his/her designated representative in written text, to ensure that each subject voluntarily participated in this experimental study and signed a written informed consent form, so that the subject's rights were fully protected, and that all the subject's information was kept strictly confidential.

The study has registered in the Chinese Clinical Trial Registry with NCT05001412.

# STUDY PLAN

- 2020.9 - 2021.1 Submissions, Ethical Review of Research Units.
- 2021.2-2022.2 The patients were enrolled and the data were recorded.
- 2022.3-2023.03 Complete follow-up and data collection of all enrolled patients.
- 2023.04-2024.12 Data analysis, paper writing and contributions.
